# Methylation of the serotonin reuptake transporter gene and non-motor symptoms in dystonia patients

**DOI:** 10.1186/s13148-022-01384-7

**Published:** 2022-12-11

**Authors:** Elze R. Timmers, Torsten Plösch, Marenka Smit, Ingrid H. Hof, Rikst Nynke Verkaik-Schakel, Marina A. J. Tijssen, Tom J. de Koning, Klary E. Niezen-Koning

**Affiliations:** 1 Department of Neurology, University Medical Center Groningen, University of Groningen, PO Box 30.001, 9700 RB Groningen, The Netherlands; 2 Expertise Center Movement Disorders Groningen, University Medical Center Groningen (UMCG), PO Box 30.001, 9700 RB Groningen, The Netherlands; 3 Department of Obstetrics and Gynecology, University Medical Center Groningen, University of Groningen, Groningen, The Netherlands; 4 Laboratory of Metabolic Diseases, Department of Laboratory Medicine, University of Groningen, University Medical Center Groningen, PO Box 30.001, 9700 RB Groningen, The Netherlands; 5 Pediatrics, Department of Clinical Sciences, Lund University, Box 117, 221 00 Lund, Sweden

**Keywords:** *SLC6A4*, Serotonin reuptake transporter, Methylation, Epigenetics, Psychiatry, Non-motor symptoms, Dystonia

## Abstract

**Background:**

Dystonia is a rare movement disorder, in which patients suffer from involuntary twisting movements or abnormal posturing. Next to these motor symptoms, patients have a high prevalence of psychiatric comorbidity, suggesting a role for serotonin in its pathophysiology. This study investigates the percentage of DNA methylation of the gene encoding for the serotonin reuptake transporter (*SLC6A4)* in dystonia patients and the associations between methylation levels and presence and severity of psychiatric symptoms.

**Methods:**

Patients with cervical dystonia (*n* = 49), myoclonus dystonia (*n* = 41) and dopa-responsive dystonia (DRD) (*n* = 27) and a group of healthy controls (*n* = 56) were included. Psychiatric comorbidity was evaluated with validated questionnaires. Methylation levels of 20 CpG sites situated 69 to 213 base pairs upstream of the start codon of *SLC6A4* were investigated. Methylation in dystonia patients was compared to healthy controls, correcting for age, and correlated with psychiatric comorbidity.

**Results:**

Bootstrapped quantile regression analysis showed that being a dystonia patient compared to a healthy control significantly explains the methylation level at two CpG sites (CpG 24: pseudo-*R*^2^ = 0.05, *p* = 0.04, CpG 32: pseudo-*R*^2^ = 0.14, *p* = 0.03). Subgroup analysis revealed that being a DRD patient significantly explained a part of the variance of methylation levels at two CpG sites (CpG 21: pseudo-*R*^2^ = 0.03, *p* = 0.00, CpG 24: pseudo-*R*^2^ = 0.06, *p* = 0.03). Regression analysis showed that methylation level at CpG 38 significantly explained a small proportion of the variance of severity score for anxiety (*R*^2^ = 0.07, *p* = 0.04) and having a diagnosis of depression (Nagelkerke *R*^2^: 0.11, *p* = 0.00). Genotype of the *5-HTTLPR* polymorphism had no additional effect on these associations.

**Conclusions:**

This study showed an association between percentage of methylation at several specific sites of the promoter region of *SLCA64* and (dopa-responsive) dystonia patients compared to healthy controls. Furthermore, methylation levels were associated with severity of anxiety and presence of a depressive disorder in the dystonia group. This study suggests alterations in the serotonergic metabolism in dystonia patients, and its relation with the non-motor symptoms.

**Supplementary Information:**

The online version contains supplementary material available at 10.1186/s13148-022-01384-7.

## Introduction

Dystonia is a hyperkinetic movement disorder in which patients not only have motor symptoms, characterized by involuntary twisting movements or abnormal posturing, but also suffer from non-motor symptoms [1]. Common non-motor symptoms are psychiatric symptoms, including but not limited to depression, anxiety and obsessive–compulsive disorder [2]. Several studies have shown that these non-motor symptoms have a serious influence on the quality of life of the patients. In some cases, non-motor symptoms are an even bigger burden than the motor symptoms [2]. Nowadays, the non-motor symptoms are considered to be an integral part of the phenotype of dystonia, but its pathophysiology is yet poorly understood.

Several neurotransmitters are thought to play a role in the pathophysiology of dystonia, including acetylcholine, dopamine and serotonin. Serotonin is a neurotransmitter of special interest because it is known to be an important regulator of mood and is, therefore, implicated to play a role in many psychiatric disorders [3, 4]. This is demonstrated by the successful use of drugs affecting the serotonergic system, such as selective serotonin reuptake inhibitors (SSRIs), in several psychiatric disorders. Also in dystonia, alterations in the serotonergic system are suggested. Low levels of the main metabolite of serotonin, 5-hydroxyindoleacetic acid, in the cerebrospinal fluid are described in several subtypes of dystonia, and in a previous study we found low levels of tryptophan, the precursor of serotonin, in blood samples of dystonia patients [5, 6].

Another way of studying the serotonergic metabolism is by investigating an important regulator of serotonin, the presynaptic reuptake transporter (5-HTT) [7]. This reuptake transporter closely controls the serotonin turnover and levels of serotonin in the synaptic cleft. The gene encoding for 5-HTT (*SLC6A4*) is a promising candidate gene for studying the pathophysiology and involvement of serotonergic metabolism in psychiatric symptoms observed in dystonia. Many studies have focused on a polymorphism (*5-HTTLPR*) in the promoter region of the *SLC6A4* gene and associations with several psychiatric disorders are reported [8–11]. It was suggested that the short allele results in less expression of 5-HTT and therefore increases the risk of having a psychiatric disorder. However, results are variable and associations between psychiatric disorders and the short allele could not be confirmed in meta-analyses [12–14]. This highlights the complex nature of the pathophysiology of psychiatric symptoms and likely relates to interactions with other genes and to environmental factors as well.

Studying epigenetic mechanisms, such as methylation, is a way to assess these environmental effects. Previous studies have shown that higher methylation levels at the promoter region of *SLC6A4* result in less expression of the gene, which can be explained by either direct blocking of transcriptional factors or by attraction of proteins which form potent repression complexes [15, 16]. It is expected that alterations in the expression of *SLC6A4* influence the serotonergic system and several studies have shown associations with methylation levels and psychiatric disorders, including depression and post-traumatic stress disorder [15, 17–20]. Some studies showed an additional effect of the *5-HTTLPR* polymorphism on developing psychiatric symptoms [15, 18]. It is important to consider the polymorphism as a potential confounder, since such polymorphisms can influence both methylation and psychiatric disorders.

The aim of this study is to investigate the percentage of DNA methylation of the promoter region of the *SLC6A4* gene in blood from large cohort of dystonia patients and compare this to healthy controls. Secondly, the association between methylation level and presence and severity of psychiatric symptoms will be assessed. Furthermore, we will determine the genotype of the *5-HTTLPR* polymorphism in dystonia patients and assess if this has an additional effect on the association with psychiatric symptoms. For this purpose, three subtypes of dystonia were included, namely idiopathic cervical dystonia (CD), dopa-responsive dystonia (DRD) and myoclonus dystonia (M-D). CD is the most common form of dystonia and starts usually by the age of 40 to 50 years, while DRD and M-D are two inherited forms with an onset in childhood. In previous studies, a high prevalence of psychiatric comorbidity was reported in all these cohorts, and alterations in serotonergic metabolism have been described [21–23]. The results from the present study add to a better understanding of the pathophysiology of the non-motor symptoms in dystonia patients which might eventually lead to better treatment and with that improvement of quality of life.

## Methods

Patients with three types of dystonia were eligible for inclusion: 1) patients with clinically confirmed idiopathic cervical dystonia, 2) patients with dopa-responsive dystonia with a confirmed *GCH1* mutation, 3) patients with myoclonus dystonia and a confirmed *SGCE* mutation. Data about the psychiatric comorbidity of these patients were published earlier [21–23]. Both adults and children (> 6 years old) were eligible to participate. Patients who received deep brain stimulation were excluded, and taking serotonergic medication was an exclusion criterion in the CD group. Informed consent was obtained from all participants, and the study was approved by the medical ethics committee of the University Medical Center Groningen (METc 2014/034).

Demographic information was collected by means of a standardized interview, and severity of dystonia was assessed by the Clinical Global Impression Severity scale (CGI-S).

### Psychiatric comorbidity

Presence and severity of psychiatric disorders in adults were assessed with, respectively, the Mini International Neuropsychiatric Interview-PLUS (MINI-PLUS), Beck Depression Inventory (BDI), Beck Anxiety Inventory (BAI) and Yale-Brown Obsessive–Compulsive Scale (Y-BOCS) [24–27]. For children, the Mini International Neuropsychiatric interview-KID (MINI-KID), Child Depression Inventory (CDI), Screen for Child Anxiety Related Emotional Disorders (SCARED) and the Children’s Yale-Brown Obsessive–Compulsive Scale (CY-BOCS) were used [28–31].

### DNA methylation analysis of SLC6A4

Methylation of 20 CpG sites situated 69 to 213 base pairs upstream of the start codon of *SLC6A4* was investigated by quantitative bisulfite pyrosequencing (Additional file 1: Fig. 1). Methylation in this region was previously identified by Zhao et al. to be associated with depressive symptoms [17].

**Fig. 1:**
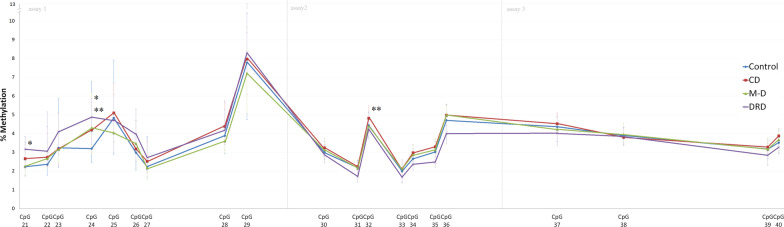
Median percentage of methylation per CpG site per group. Error bars represent interquartile range. Distance between the CpG sites on the x-axis represents the distance of CpG sites in the gene. *Indicates that variance of methylation level at this CpG site is significantly predicted by having dopa-responsive dystonia compared to healthy controls when corrected for age using bootstrapped quantile regression analysis.**Indicates that the variance of methylation at this CpG site is significantly predicted by having dystonia compared to healthy controls, when corrected for age. *CD* cervical dystonia, *M-D* myoclonus dystonia, *DRD* dopa-responsive dystonia

Venous blood samples were collected from all participants. DNA was isolated from whole blood (Qiagen QIAamp DNA microkit no. 56304 and Wizard Genomic DNA Purification Kit; Promega, no. A1125) and was treated with bisulfite to convert unmethylated C residues to uracil residues, whereas methylated C nucleotides are protected using the Zymo EZ DNA Methylation-Gold™ Kit. PCR primers were created for CpG sites 21 till 40 as described by Riese et al. [32], and details can be found in Additional file 1: Table 13. PCR products were validated by agarose gel electrophoresis. DNA methylation was then analyzed on a PyroMark Q24 Pyrosequencer (Qiagen) to obtain percentages of methylation per CpG site.

### Genotype of 5-HTTLPR

The presence of three different variants of *5-HTTLPR* (*L*_A_, *L*_G_ and *S*) was tested. These alleles are known to be associated with different transcriptional activities of *SLC6A4*; the *L*_A_ allele causes high transcriptional activity, while patients with the *L*_G_ and *S* allele have low transcriptional activity [33]. Detailed information about the methodology can be found in Additional file 1.

### Statistics

Statistical analysis was performed using IBM SPSS Statistics version 28 and STATA/SE 17. A *p* value < 0.05 was considered statistically significant.

One-way ANOVA or Kruskal–Wallis tests were used to compare continuous clinical characteristics including severity of psychiatric symptoms between the four groups. Clinical characteristics of all dystonia patients together were compared to the healthy controls using Student’s t tests or Mann–Whitney U tests. Dichotomous variables were compared between the groups using *χ*^2^ tests.

Kruskal–Wallis and Mann–Whitney U tests were used to compare methylation levels (including mean methylation) between the four groups or dystonia vs. controls, respectively. Spearman’s correlation analysis was used to assess associations between age and methylation in all participants. To correct for the possible confounding effect of age, a bootstrapped quantile (median) regression analysis was performed with methylation levels as dependent variable and ‘age’ and ‘group (decoded as 3 dummy variables, with controls as reference group)’ or ‘having dystonia,’ respectively, as independent variables. This quantile regression analysis is robust for outliers and the non-normal distribution of the methylation levels. Analyses were also performed for male and female participants separately.

To assess association between severity and presence of psychiatric comorbidity and methylation levels in the whole dystonia group, Spearman’s correlation analysis and Mann–Whitney U tests were performed. To be able to combine the severity rating scales of children and adults, a z-score was computed based on the control group. The above-mentioned analyses were also performed for each dystonia subtype. Variables that showed a significant association with methylation levels on a specific CpG site were entered into an ordinary least squares or logistic regression analysis. First, age and severity of dystonia were entered as covariates to evaluate confounding effects. Next, regression analyses were performed with addition of the *5-HTTLPR* genotype. Assumptions of regression analysis were checked, and results for the ordinary least squares regression were adjusted for heteroskedasticity according to Hayes et al. [34]. To further examine the effect of the *5-HTTLPR* polymorphism on psychiatry, Kruskal–Wallis tests and *χ*^2^ tests were performed.

## Results

We included a total of 195 participants. No blood samples could be obtained of 20 participants (1 CD, 11 M-D, 2 DRD and 6 healthy controls), and because of technical issues of the pyrosequencing, no reliable results were produced for one CD patient. This resulted in 49 CD, 41 M-D, 27 DRD patients and 56 healthy controls, both children and adults, to be included in the final analysis.

An overview of demographic features, genotype of the *5-HTTLPR* polymorphism, motor symptoms and psychiatric comorbidity can be found in Table 1. Age was significantly different between the four groups (*p* = 0.00), with younger participants in the DRD (37 years) and M-D (44 years) group, compared to the CD patients (54 years) and healthy controls (50 years). Relatively more patients with DRD (44%) were homozygous for the *L*_A_ allele compared to the other dystonia groups (CD: 14%, M-D: 29%) and healthy controls (25%).

**Table 1:** Demographics and psychiatric comorbidity of participants

	CD (*n* = 49)	M-D (*n* = 41)	DRD (*n* = 27)	Controls (*n* = 56)	*p-*value^1^	Dystonia (*n* = 117)	*p* value^2^
Age (SD)	54 (11)	44 (20)	37 (22)	50 (17)	***0.00***^***a***^	47 (19)	0.27^b^
Gender M	14 (29%)	17 (42%)	9 (33%)	16 (29%)	*0.52*^*c*^	40 (34%)	0.46^c^
Genotype							
*L*_A_/*L*_A_	7 (14%)	12 (29%)	12 (44%)	14 (25%)	***0.03***^***c***^	31 (27%)	0.79^c^
*S*/*S*	11 (22%)	4 (10%)	5 (19%)	10 (18%)	*0.58*^*c*^	20 (17%)	0.94^c^
*L*_A_/*S*	25 (51%)	13 (32%)	7 (26%)	21 (38%)	*0.16*^*c*^	45 (39%)	0.84^c^
*L*_A_/*L*_G_	5 (10%)	4 (10%)	2 (7%)	6 (11%)	*0.96*^*c*^	11 (9%)	0.81^c^
*S*/*L*_G_	1 (2%)	3 (7%)	1 (4%)	4 (7%)	*0.54*^*c*^	5 (4%)	0.44^c^
*L*_G_/*L*_G_	0	1 (2%)	0	0	*0.31*^*c*^	1 (1%)	0.48^c^
CGI (range)	4 (2–7)	3 (1–5)	2 (1–3)		***0.00***^***d***^		
Duration dystonia in years (range)	10 (1–52)	47 (7–73)	23 (1–71)		***0.00***^***d***^		
BDI (range)*	8 (0–28)	8 (0–30)	5 (0–18)	3 (0–19)	***0.00***^***d***^	8 (0–30)	**0.00**^**e**^
BAI (range)*	8 (1–31)	8 (0–42)	6 (0–12)	3 (0–21)	***0.00***^***d***^	7 (0–42)	**0.00**^**e**^
CDI (range)**	NA	101 (82–126)	88 (73–117)	98 (84–103)	*0.28*^*d*^	89 (73–126)	0.75^e^
SCARED (range)**	NA	4 (1–12)	6 (2–19)	2 (1–24)	*0.40*^*d*^	4 (1–19)	0.27^e^
(C)Y-BOCS (range)	0 (0–12)	0 (0–12)	0 (0–18)	0 (0–9)	*0.10*^*d*^	0 (0–18)	**0.03**^**e**^
Psychiatric diagnosis	33 (67%)	24 (59%)	17 (63%)	17 (30%)	***0.00***^***c***^	74 (63%)	**0.00**^**c**^
Depressive disorder	16 (33%)	13 (32%)	9 (33%)	8 (14%)	*0.09*^*c*^	38 (33%)	**0.01**^**c**^
Anxiety disorder	22 (45%)	20 (49%)	12 (44%)	5 (9%)	***0.00***^***c***^	54 (46%)	**0.00**^**c**^

Values are given as mean (SD), frequency (%), or median (range) as indicated. ^1^*p* values of statistical comparison between the four groups, significant differences are depicted in bold-italics, ^2^*p* values of statistical comparison of all dystonia patients versus controls, significant differences are depicted in bold. *questionnaires only completed by adults (age > 17 years), ** questionnaires only completed by children (7 M-D, 10 DRD patients and 5 healthy controls. The following tests are used to compute *p* values: ^a^one-way ANOVA, ^b^Student’s t test, ^c^χ^2^ test, ^d^Kruskal–Wallis, ^e^Mann–Whitney U test. *CD* cervical dystonia, *M-D* myoclonus-dystonia, *DRD* dopa-responsive dystonia, *BDI* Beck Depression Inventory, *BAI* Beck Anxiety Inventory, *CDI* Child Depression Inventory, *SCARED* Screen for Child Anxiety Related Emotional Disorders, *(C)Y-BOCS* (Child) Yale-Brown Obsessive–Compulsive Scale

Participants used various types of medication; important to mention is the levodopa use in 21 of the 27 DRD patients. Of the remaining six DRD patients four were asymptomatic, one ceased treatment because of side effects, and one patient felt that the effort of taking daily medication did not outweigh the benefits. Some patients also used SSRIs (in 2 DRD and 5 M-D patients) or selective noradrenaline reuptake inhibitors (2 M-D patients) or tricyclic anti-depressives (2 DRD patients). Taking serotonergic medication was an exclusion criterion in the CD group. Motor symptoms were treated successfully in the MD group with clonazepam (*n* = 6), trihexyphenidyl (*n* = 3), botulinum toxin (*n* = 3), levetiracetam (*n* = 2) and valproate acid (*n* = 1). Almost all CD patients (*n* = 47) received botulinum toxin injections. No patients were treated with deep brain stimulation.

Patients with dystonia had significantly more often a lifetime diagnosis of a psychiatric disorder, according to the MINI-PLUS/KID, compared to controls and adults scored higher on psychiatric severity scales (Table 1). More details about the psychiatric symptoms in this cohort can be found in our earlier publications [21–23]. No association between presence or severity of the psychiatric symptoms and severity of motor symptoms was found.

### DNA methylation analysis of SLC6A4

Median methylation levels per group are shown in Fig. 1. Analyses without a correction for age showed a significant difference between all dystonia patients and healthy controls at CpG 24 (4.3% vs. 3.2%, *p* = 0.03). Significant differences between the four groups at CpG 32 (*p* = 0.04), 34 (*p* = 0.04), 35 and 36 (both *p* = 0.02) were found (Additional file 1: Table 1). Age was significantly correlated with CpG 24 and CpG 29 t/m 40 (Additional file 1: Table 2).

**Table 2:** Regression analysis with severity z-score of depression or anxiety or having a lifetime depressive disorder as dependent variables

Model	Variables	*R*^2^	*B*	SE	*p* value
**Linear regression; z-score depression severity**					
1	CpG34	0.04	0.49	0.25	0.06
					0.06
**Linear regression; z-score anxiety severity**					
1	CpG34	0.04	0.53	0.29	0.07
					0.07
1	CpG38	0.07			**0.04**
			− 0.71	0.34	**0.04**
2		0.16			**0.00**
	CpG38		− 0.55	0.88	**0.01**
	Age		0.03	0.21	**0.00**
	CGI		0.18	0.01	0.13
3		0.07			**0.03**
	CpG38		− 0.66	1.77	0.08
	Genotype*:				
	*L*_A_/*L*_A_				
	*S*/*S*		0.03	0.63	0.96
	Other		0.45	0.46	0.33

Model	Variables	Nagelkerke *R*^2^	*B*	SE	*p* value
**Logistic regression; Lifetime presence of Depression**					
1		0.11			**0.00**
	CpG38		0.95	0.34	**0.01**
2		0.14			**0.01**
	CpG38		0.96	0.36	**0.01**
	Age		− 0.02	0.01	0.21
	CGI		− 0.15	0.15	0.29
3		0.11			**0.04**
	CpG38		0.89	0.34	**0.01**
	Genotype*:				
	*L*_A_/*L*_A_				
	*S*/*S*		0.35	0.68	0.60
	Other		0.07	0.02	0.88

Significant differences are depicted in bold

Multiple regression analyses using ordinary least squares method with correction for heteroskedasticity and logistic regression analysis were performed. *Genotype was entered as dummy variables with *L*_A_/*L*_A_ as a reference. ‘Other’ genotype means either a *L*_A_/*S*, *L*_A_/*L*_G_, *S*/*L*_G_ or *L*_G_/*L*_G_ genotype

Bootstrapped quantile regression analysis with correction for age showed that being a dystonia patient compared to a healthy control significantly explains the methylation level at CpG 24 (pseudo-*R*^2^ = 0.05, *B* = 1.12, *p* = 0.04) and CpG 32 (pseudo-*R*^2^ = 0.14, *B* = 0.37, *p* = 0.03) (Fig. 1, Additional file 1: Table 3). Furthermore, being a DRD patient compared to a healthy control, with correction for age, significantly explained a part of the variance of methylation level at CpG 21 (pseudo-*R*^2^ = 0.03, *B* = 1.02, *p* = 0.00) and CpG 24 (pseudo-*R*^2^ = 0.06, *B* = 1.73, *p* = 0.03) (Fig. 1, Additional file 1: Table 4). In female participants, results were similar, with an additional finding of significant involvement of the CD group in the model of methylation level at CpG 21 and 24 and the DRD group of methylation at CpG 23 (Additional file 1: Tables 5 and 6). In males, quantile regression analysis showed no significant findings (Additional file 1: Tables 7 and 8).

### Association with psychiatric comorbidity in dystonia patients

**Fig. 2:**
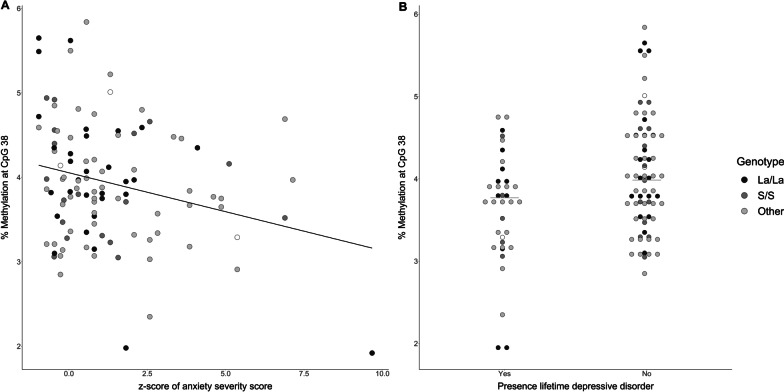
Associations between psychiatry and methylation at CpG38 in all dystonia patients

Spearman’s correlation analysis showed significant positive correlations between methylation levels at CpG 34 and severity score of depression (*r*_s_ = 0.23, *p* = 0.02), and anxiety (*r*_s_ = 0.28, *p* = 0.00), and a negative correlation with methylation level at CpG 38 and severity of anxiety (*r*_s_ =  − 0.22, *p* = 0.04) (Fig. 2 and Additional file 1: Table 9). Stepwise ordinary least squares regression analysis showed that methylation level at CpG 38 significantly explained a small proportion of the variance of severity score for anxiety (*R*^2^ = 0.07, *p* = 0.04). Addition of age and severity of dystonia to the model significantly increased the model (Δ*R*^2^ = 0.09, *p* = 0.00) (Table 2). Genotype of the *5-HTTLPR* polymorphism did not significantly contribute to the model.

Mann–Whitney U tests assessing methylation level in dystonia patients with or without psychiatric comorbidity showed a significant difference at CpG 28 (*p* = 0.04); however, this is most likely due to two outliers (Additional file 1: Fig. 1). Dystonia patients with a depressive disorder in their lifetime had a lower percentage of methylation at CpG 38 (*p* = 0.01) (Fig. 2B). Logistic regression showed, similar to the linear regression, a significant model with percentage methylation level at CpG 38 as independent variable and having a diagnosis of depression as dependent variable (Nagelkerke *R*^2^: 0.11, *p* = 0.00). Addition of age and severity of dystonia and subsequently genotype did not significantly change the model (Table 2).

Results per dystonia phenotype can be found in the supplementary material (Additional file 1: Tables 10 and 11). CpG sites at which a significant association with psychiatry was found differed slightly between phenotypes; however, no characteristic methylation pattern for each subgroup was found. Genotype of the *5-HTTLPR* polymorphism was not related to psychiatry severity scores or having a lifetime psychiatric disorder (Additional file 1: Table 13).

## Discussion

This study shows an association between percentage of methylation at several specific sites of the promoter region of *SLCA64* and dystonia patients (CD, DRD and M-D) compared to healthy controls. Furthermore, an association between methylation level and severity of anxiety and presence of a depressive disorder was found in the dystonia group.

Important regulators of synaptic serotonergic metabolism are the serotonin reuptake transporter and its gene (*SLC6A4)*. The expression of *SLC6A4* is, in turn, regulated by both genetic and epigenetic factors, such as the 5-*HTTLPR* polymorphism and methylation [15, 35, 36]. Our results showed that in dystonia patients, and especially DRD patients, an association was present with, a relatively higher, methylation level at some CpG sites. This association was found in only two of the 20 sites that were investigated. Our findings do indicate that methylation is altered in dystonia patients. This may lead to an altered serotonergic system, which is in line with previous literature. A review by Smit et al. showed evidence for involvement of the serotonergic metabolism in dystonia, including altered metabolites of serotonin in the cerebral spinal fluid [5]. DRD, the dystonia cohort that predicted methylation level most, is a dystonia subtype of special interest, since these patients have a mutation in one of the enzymes in the mono-amine pathway leading to impairments in the production of both dopamine and serotonin [37]. This is highly suggestive of an altered serotonergic state in patients with DRD, which is supported by our findings. Alterations in methylation patterns can be the result of environmental factors, such as the extensively studied effect of early life stress on methylation of *SLC6A4* [38], but genetic factors might play a role as well [39, 40]. To the best of our knowledge, no studies are performed investigating the effect of mutations in the mono-amine pathway (such as the *GCH1* mutation) on methylation of the *SLC6A4* gene.

In this study, we investigated methylation levels in blood samples and not in the area of interest, namely the brain. However, an earlier study by our group showed that the methylation levels at the CpG sites that we investigated in blood were highly correlated with the methylation levels in postmortem amygdala tissue, an important structure in the regulation of emotional responses [32]. Furthermore, previous studies showed an association between higher methylation levels in the promoter region of *SLC6A4* in peripheral blood samples and lower expression of the serotonin reuptake transporter in the prefrontal cortex and lower positron emission tomography (PET) measurements of serotonin synthesis in orbitofrontal cortex [41, 42]. Other imaging studies reported an association between methylation levels in blood samples and altered connectivity in fronto-limbic areas, suggesting altered emotional processing [43]. Together, these studies show that measurement of methylation in peripheral samples provides functionally relevant information about brain regions involved in the pathophysiology of psychiatric disorders.

The main reason why serotonin is thought to be implicated in dystonia is the high prevalence of non-motor symptoms, such as psychiatric symptoms, in dystonia patients. In several previous studies, associations between methylation of the *SLC6A4* gene and psychiatric disorders are reported [15, 17–20]. The specific CpG sites at which we found an association between dystonia and methylation were, however, not correlated with the outcomes on the psychiatry questionnaires. Nevertheless, we did find an association between lower methylation at another CpG site (CpG 38) and severity of anxiety and the presence of a lifetime diagnosis of depression. To the best of our knowledge, this study reports for the first time an association between the severity of anxiety and methylation of *SLC6A4.* Only one previous study investigated the methylation levels of *SLC6A4* in patients with an anxiety disorder, namely panic disorder. They did not find altered methylation levels in patients with a panic disorder, but did found changes in patients with a comorbid major depressive disorder [44].

The literature is not conclusive on how methylation of *SLC6A4* differs in depression. Our findings are in contrast to the study by Philibert et al. who found a trend for an association between increased overall methylation and a lifetime history of major depressive disorder [15]. Other studies have shown as well that a higher methylation levels were associated with depression or severity of depressive symptoms [45, 46]. On the other hand, a study by Okada et al. did not find any differences in methylation between patients with a depressive disorder and controls [47]. These seemingly contrasting findings might be explained by the different CpG sites that have been investigated and different clinical characteristics of and/or environmental factors in the cohorts. This is the first time that the relationship between methylation of the *SLC6A4* gene and depressive symptoms was investigated in a dystonia cohort. Previous studies were performed in patients with purely a psychiatric disorder, while our patients have psychiatric symptoms in addition to suffering from dystonia. This likely explains the contrasting findings.

In the study by Zhao et al., who investigated the same CpG sites, the CpG site at which we found associations with psychiatry (CpG 3, CpG 38 in our study) was not significantly correlated with severity of depression (*p* = 0.051) in a group male twin veterans, but in 10 other investigated CpG sites they did found a significant correlation with severity of depression [17]. This slightly different findings might be the result of the different characteristics of our cohorts, with our cohort predominantly consisting of females. Previous studies have shown associations between methylation levels of the *SLC6A4* gene and gender, and some even show that gender influences the association between methylation and psychiatry [15, 48–50]. In line with this, in our cohort the analysis in female participants only showed more significant associations between methylation and dystonia than in males.

Our results showed no association between the 5-*HTTLPR* polymorphism and psychiatry. Furthermore, the polymorphism did not seem to have an influence on the relationship between methylation and psychiatry. Our findings are in line with the study by Zhao et al. who investigated the same CpG sites [17]. On the other hand, other studies did show an effect of the polymorphism on the relationship between methylation level and psychiatry [15, 18, 48], with mainly carriers of the *S*-allele being more prone in developing psychiatric disorders. Again, the different CpG sites our cohorts that were investigated in these studies might be responsible for these contrasting findings.

An earlier study in *KMT2B-*related dystonia studied the methylation pattern on a genome-wide scale and found a ‘episignature’ of 113 CpG sites with increased methylation [51]. This genome-wide analyses might provide useful information about the pathophysiology of dystonia, and it would be interesting to perform such analysis on other subtypes of dystonia, including DRD, MD and CD. In this study, we used a candidate gene approach since we hypothesized a role for serotonin in the pathophysiology of, especially the non-motor symptoms, dystonia. As previously mentioned, the association between methylation of *SLC6A4* and psychiatric symptoms is widely studied and our results indeed point toward involvement of the serotonergic system. Further studies are necessary to confirm our findings.

This research has some limitations. First, age was significantly different between the groups, which might be a confounding factor since it is known that methylation patterns change during lifetime, and in our cohort, methylation levels were positively correlated with age. Although we corrected for age in our analysis, it cannot completely rule out that it had no effect on our results. The motor symptoms of the patients were adequately under control with pharmacological treatment. However, the medication use of some of the participants might have had some influence on our results; especially the use of antidepressant treatment of some of the patients might have influenced the methylation as was reported in previous studies [47].

## Conclusions

Concluding, this study showed for the first time the role of *SLC6A4* and non-motor symptoms in a relatively large cohort of dystonia patients. We found an association between (dopa-responsive) dystonia and methylation levels at specific CpG sites in the promoter region of *SLC6A4*. Furthermore, correlations between psychiatric questionnaires and methylation levels were found, which is in line with previous literature in other cohorts. Our results suggest alterations in the serotonergic metabolism in dystonia patients and its role in the non-motor symptoms. Further studies have to replicate and verify our findings, and this will lead to a better understanding of the pathophysiology of non-motor symptoms in dystonia.

## Supplementary Information

**Additional file 1**. Supplementary tables and figures.

## Data Availability

The datasets used and/or analyzed during the current study are available from the corresponding author on reasonable request.

## Abbreviations

5-HTT: Serotonin reuptake transporter
BAI: Beck Anxiety Inventory
BDI: Beck Depression Inventory
CD: Cervical dystonia
CDI: Child Depression Inventory
CpG: Cytosine nucleotide followed by a guanine nucleotide
CY-BOCS: Children’s Yale-Brown Obsessive–Compulsive Scale
DRD: Dopa-responsive dystonia
M–D: Myoclonus dystonia
MINI-KID: Mini International Neuropsychiatric interview-KID
MINI-PLUS: Mini International Neuropsychiatric Interview-PLUS
PET: Positron emission tomography
SCARED: Screen for Child Anxiety Related Emotional Disorders
SSRI: Selective serotonin reuptake inhibitor
Y-BOCS: Yale-Brown Obsessive–Compulsive Scale

## References

[1] Albanese A (2013). Phenomenology and classification of dystonia: a consensus update. Mov Disord.

[2] Kuyper DJ, Parra V, Aerts S, Okun MS, Kluger BM (2011). Nonmotor manifestations of dystonia: a systematic review. Mov Disord.

[3] Berger M, Gray JA, Roth BL (2009). The expanded biology of serotonin. Annu Rev Med.

[4] Lin S-H, Lee L-T, Yang YK (2014). Serotonin and mental disorders: a concise review on molecular neuroimaging evidence. Clin Psychopharmacol Neurosci.

[5] Smit M (2016). Serotonergic perturbations in dystonia disorders—a systematic review. Neurosci Biobehav Rev.

[6] Timmers ER (2021). Dopaminergic and serotonergic alterations in plasma in three groups of dystonia patients. Parkinsonism Relat Disord.

[7] Fidalgo S, Ivanov DK, Wood SH (2013). Serotonin: from top to bottom. Biogerontology.

[8] Lesch K-P (1998). Review : serotonin transporter and psychiatric disorders: listening to the gene. Neuroscientist.

[9] Lonsdorf TB (2009). The symptomatic profile of panic disorder is shaped by the 5-HTTLPR polymorphism. Prog Neuropsychopharmacol Biol Psychiatry.

[10] Caspi A (2003). Influence of life stress on depression: moderation by a polymorphism in the 5-HTT gene. Science.

[11] Lesch K-P (1996). Association of anxiety-related traits with a polymorphism in the serotonin transporter gene regulatory region. Science.

[12] Majumdar A, Patel P, Pasaniuc B, Ophoff RA (2021). A summary-statistics-based approach to examine the role of serotonin transporter promoter tandem repeat polymorphism in psychiatric phenotypes. Eur J Hum Genet.

[13] Culverhouse RC (2018). Collaborative meta-analysis finds no evidence of a strong interaction between stress and 5-HTTLPR genotype contributing to the development of depression. Mol Psychiatry.

[14] Mak L, Streiner DL, Steiner M (2015). Is serotonin transporter polymorphism (5-HTTLPR) allele status a predictor for obsessive-compulsive disorder? A meta-analysis. Arch Womens Ment Health.

[15] Philibert RA (2008). The relationship of 5HTT (SLC6A4) methylation and genotype on mRNA expression and liability to major depression and alcohol dependence in subjects from the Iowa Adoption Studies. Am J Med Genet B Neuropsychiatr Genet.

[16] Mazzio EA, Soliman KFA (2012). Basic concepts of epigenetics: impact of environmental signals on gene expression. Epigenetics.

[17] Zhao J, Goldberg J, Bremner JD, Vaccarino V (2013). Association between promoter methylation of serotonin transporter gene and depressive symptoms: a monozygotic twin study. Psychosom Med.

[18] Lam D (2018). Genotype-dependent associations between serotonin transporter gene (SLC6A4) DNA methylation and late-life depression. BMC Psychiatry.

[19] Koenen KC (2011). SLC6A4 methylation modifies the effect of the number of traumatic events on risk for posttraumatic stress disorder. Depress Anxiety.

[20] Sugawara H, Bundo M, Ishigooka J, Iwamoto K, Kato T (2013). Epigenetic regulation of serotonin transporter in psychiatric disorders. J Genet Genom.

[21] Timmers ER (2017). Non-motor symptoms and quality of life in dopa-responsive dystonia patients. Parkinsonism Relat Disord.

[22] Smit M (2016). Psychiatric co-morbidity is highly prevalent in idiopathic cervical dystonia and significantly influences health-related quality of life: Results of a controlled study. Parkinsonism Relat Disord.

[23] Timmers ER (2019). Myoclonus-dystonia: distinctive motor and non-motor phenotype from other dystonia syndromes. Parkinsonism Relat Disord.

[24] Sheehan DV, et al. The Mini-International Neuropsychiatric Interview (M.I.N.I.): the development and validation of a structured diagnostic psychiatric interview for DSM-IV and ICD-10. J Clin Psychiatry 59 Suppl 20, 22–33;quiz 34–57 (1998). 9881538

[25] Beck AT, Steer RA, Carbin MG (1988). Psychometric properties of the Beck Depression Inventory: twenty-five years of evaluation. Clin Psychol Rev.

[26] Beck AT, Epstein N, Brown G, Steer RA (1988). An inventory for measuring clinical anxiety: psychometric properties. J Consult Clin Psychol.

[27] Goodman WK, et al. The Yale-Brown Obsessive Compulsive Scale. I. Development, use, and reliability. Arch Gen Psychiatry 46, 1006–1011. 10.1001/archpsyc.1989.01810110048007 (1989). 10.1001/archpsyc.1989.01810110048007 2684084

[28] Scahill L (1997). Children's Yale-Brown obsessive compulsive scale: reliability and validity. J Am Acad Child Adolesc Psychiatry.

[29] Sheehan DV (2010). Reliability and validity of the Mini International Neuropsychiatric Interview for Children and Adolescents (MINI-KID). J Clin Psychiatry.

[30] Kovacs M (1985). The Children's Depression, Inventory (CDI). Psychopharmacol Bull.

[31] Birmaher B (1997). The Screen for Child Anxiety Related Emotional Disorders (SCARED): scale construction and psychometric characteristics. J Am Acad Child Adolesc Psychiatry.

[32] Riese H, et al. Association between methylation of the SLC6A4 promoter region in peripheral blood leukocytes and methylation in amygdala tissue. Psychosom Med 2014;76. 10.1097/PSY.0000000000000048 24632895

[33] Nakamura M, Ueno S, Sano A, Tanabe H (2000). The human serotonin transporter gene linked polymorphism (5-HTTLPR) shows ten novel allelic variants. Mol Psychiatry.

[34] Hayes AF, Cai L (2007). Using heteroskedasticity-consistent standard error estimators in OLS regression: an introduction and software implementation. Behav Res Methods.

[35] Hu X-Z (2006). Serotonin transporter promoter gain-of-function genotypes are linked to obsessive-compulsive disorder. Am J Hum Genet.

[36] Olsson CA (2010). Prospects for epigenetic research within cohort studies of psychological disorder: a pilot investigation of a peripheral cell marker of epigenetic risk for depression. Biol Psychol.

[37] Wijemanne S, Jankovic J (2015). Dopa-responsive dystonia–clinical and genetic heterogeneity. Nat Rev Neurol.

[38] Provenzi L, Giorda R, Beri S, Montirosso R (2016). SLC6A4 methylation as an epigenetic marker of life adversity exposures in humans: a systematic review of literature. Neurosci Biobehav Rev.

[39] Gaunt TR (2016). Systematic identification of genetic influences on methylation across the human life course. Genome Biol.

[40] Alameda L, et al. Can epigenetics shine a light on the biological pathways underlying major mental disorders? Psychol Med. 10.1017/S0033291721005559 (2022). 10.1017/S0033291721005559 PMC9280283 35193719

[41] Drabe M (2017). Serotonin transporter gene promoter methylation status correlates with in vivo prefrontal 5-HTT availability and reward function in human obesity. Transl Psychiatry.

[42] Wang D (2012). Peripheral SLC6A4 DNA methylation is associated with in vivo measures of human brain serotonin synthesis and childhood physical aggression. PLoS ONE.

[43] Ismaylova E (2018). Serotonin transporter promoter methylation in peripheral cells and neural responses to negative stimuli: a study of adolescent monozygotic twins. Transl Psychiatry.

[44] Schiele MA (2019). Hypermethylation of the serotonin transporter gene promoter in panic disorder—Epigenetic imprint of comorbid depression?. Eur Neuropsychopharmacol.

[45] Kim JM (2013). A longitudinal study of SLC6A4 DNA promoter methylation and poststroke depression. J Psychiatr Res.

[46] Iga J (2016). Association study of polymorphism in the serotonin transporter gene promoter, methylation profiles, and expression in patients with major depressive disorder. Hum Psychopharmacol.

[47] Okada S (2014). The potential of SLC6A4 gene methylation analysis for the diagnosis and treatment of major depression. J Psychiatr Res.

[48] Beach SRH, Dogan MV, Brody GH, Philibert RA (2014). Differential impact of cumulative SES risk on methylation of protein–protein interaction pathways as a function of SLC6A4 genetic variation in African American young adults. Biol Psychol.

[49] Booij L (2015). DNA methylation of the serotonin transporter gene in peripheral cells and stress-related changes in hippocampal volume: a study in depressed patients and healthy controls. PLoS ONE.

[50] Sanwald S (2021). Factors related to age at depression onset: the role of SLC6A4 methylation, sex, exposure to stressful life events and personality in a sample of inpatients suffering from major depression. BMC Psychiatry.

[51] Mirza-Schreiber N (2021). Blood DNA methylation provides an accurate biomarker of KMT2B-related dystonia and predicts onset. Brain.

