# Supplementary material for: Methylation of the serotonin reuptake transporter gene and non-motor symptoms in dystonia patients
Source: Clin Epigenetics. 2022 Dec 11;14:170. doi: 10.1186/s13148-022-01384-7 (PMC9743677; doi:10.1186/s13148-022-01384-7)
Supplement: Supplementary file 1 — Additional file 1. Supplementary tables and figures. [file 13148_2022_1384_MOESM1_ESM.docx]

|  | **CD**  **(n=49)** | **M-D**  **(n=41)** | **DRD**  **(n=27)** | **Controls**  **(n=56)** | **p-value^1^** | **Dystonia (n= 117)** | **p-value^2^** |
| --- | --- | --- | --- | --- | --- | --- | --- |
| **CpG 21** | 2.7 (1.4)^a^ | 2.3 (1.3)^e^ | 3.2 (1.8)^m^ | 2.2 (1.9) | *0.16* | 2.6 (1.5)^w^ | 0.35 |
| **CpG 22** | 2.8 (1.9)^a^ | 2.7 (1.5)^e^ | 3.1 (3.1)^m^ | 2.4 (2.7)^p^ | *0.64* | 2.8 (2.0)^w^ | 0.35 |
| **CpG 23** | 3.2 (3.3)^a^ | 3.2 (2.1)^e^ | 4.1 (2.3) | 3.3 (3.9)^p^ | *0.42* | 3.3 (2.6)^u^ | 0.96 |
| **CpG 24** | 4.2 (3.3) | 4.3 (3.6)^e^ | 4.9 (2.6)^m^ | 3.2 (4.7)^p^ | *0.17* | 4.3 (2.9)^u^ | **0.03** |
| **CpG 25** | 5.1 (3.2) | 4.1 (2.6)^e^ | 4.7 (3.3)^l^ | 4.9 (5.1)^p^ | *0.54* | 4.7 (2.9)^w^ | 0.96 |
| **CpG 26** | 3.2 (2.9)^b^ | 3.5 (2.3)^e^ | 4.0 (2.9)^m^ | 3.0 (2.7)^p^ | *0.44* | 3.5 (2.6)^x^ | 0.21 |
| **CpG 27** | 2.5 (1.6)^c^ | 2.2 (1.8)^f^ | 2.7 (1.8)^l^ | 2.2 (2.3)^p^ | *0.47* | 2.5 (1.7)^y^ | 0.83 |
| **CpG 28** | 4.4 (2.8)^d^ | 3.6 (1.8)^g^ | 4.2 (2.1)^n^ | 3.9 (2.9)^c^ | *0.16* | 4.0 (2.1)^z^ | 0.97 |
| **CpG 29** | 8.0 (3.5)^d^ | 7.2 (5.0)^h^ | 8.3 (7.3)^o^ | 7.8 (6.4)^c^ | *0.53* | 8.0 (4.3)^aa^ | 0.95 |
| **CpG 30** | 3.2 (1.0)^a^ | 3.1 (1.3)^g^ | 2.9 (1.0)^n^ | 3.0 (1.0)^a^ | *0.35* | 3.1 (1.1)^ab^ | 0.75 |
| **CpG 31** | 2.3 (0.6)^a^ | 2.2 (0.9)^i^ | 1.7 (1.2)^n^ | 2.2 (0.9)^q^ | *0.36* | 2.2 (0.8)^ac^ | 0.78 |
| **CpG 32** | 4.8 (1.1)^a^ | 4.5 (1.2)^j^ | 4.3 (1.7)^n^ | 4.5 (1.4)^r^ | ***0.04*** | 4.6 (1.2)^ad^ | 0.12 |
| **CpG 33** | 2.1 (0.9)^a^ | 2.1 (0.7)^j^ | 1.7 (0.8)^l^ | 2.0 (1.0)^s^ | *0.09* | 2.1 (0.9)^ae^ | 0.36 |
| **CpG 34** | 3.0 (1.0)^a^ | 2.9 (0.9)^j^ | 2.4 (1.0)^l^ | 2.7 (0.9)^s^ | ***0.04*** | 2.8 (1.0)^ae^ | 0.26 |
| **CpG 35** | 3.3 (0.9)^a^ | 3.1 (1.5)^k^ | 2.5 (1.1)^l^ | 3.0 (1.1)^s^ | ***0.02*** | 3.1 (1.1)^af^ | 0.57 |
| **CpG 36** | 5.0 (1.2)^a^ | 5.0 (1.2)^k^ | 4.0 (1.7)^l^ | 4.8 (1.7)^s^ | ***0.02*** | 4.9 (1.3)^af^ | 0.84 |
| **CpG 37** | 4.5 (1.1)^b^ | 4.3 (1.2)^k^ | 4.0 (1.6)^l^ | 4.4 (1.4)^r^ | *0.17* | 4.4 (1.3)^ae^ | 0.77 |
| **CpG 38** | 3.8 (0.9)^a^ | 4.0 (1.1)^k^ | 3.9 (1.0) | 3.9 (0.9)^s^ | *0.93* | 3.9 (1.0)^y^ | 0.72 |
| **CpG 39** | 3.3 (0.7)^a^ | 3.2 (1.2)^k^ | 2.9 (1.4) | 3.2 (1.0)^t^ | *0.29* | 3.2 (1.0)^y^ | 0.88 |
| **CpG 40** | 3.9 (1.0)^a^ | 3.7 (1.2)^k^ | 3.3 (0.9) | 3.5 (1.2)^t^ | *0.06* | 3.7 (1.1)^y^ | 0.40 |
| **Mean** | 3.8 (1.0) | 3.9 (1.0) | 3.8 (0.9) | 3.7 (1.6) | *0.59* | 3.8 (0.9) | 0.76 |

**Supplementary material**

Supplementary table 1. Methylation of the promotor region of *SLC6A4* in different dystonia groups uncorrected for age. Data is presented as median percentage of methylation (interquartile range). Kruskal Wallis^1^ and Mann Whitney U tests^2^ were used to compute p-values. Due to technical errors we did not retrieve reliable results for some samples, the number of samples used in the analyses are: ^a^48, ^b^47, ^c^46, ^d^41, ^e^40, ^f^39, ^g^32, ^h^36, ^i^33, ^j^34, ^k^35, ^l^25, ^m^26, ^n^24, ^o^22, ^p^55, ^q^49, ^r^51, ^s^52, ^t^53, ^u^115, ^v^116, ^w^114, ^x^113, ^y^110, ^z^97, ^aa^99, ^ab^104, ^ac^105, ^ad^106, ^ae^107, ^af^108

|  | Age |  |  |
| --- | --- | --- | --- |
|  | Correlation Coefficient | *p-*value | N |
| CpG21 | 0.07 | 0.34 | 170 |
| CpG22 | 0.07 | 0.37 | 169 |
| CpG23 | -0.03 | 0.69 | 170 |
| CpG24 | 0.16 | **0.04** | 170 |
| CpG25 | 0.09 | 0.27 | 169 |
| CpG26 | 0.06 | 0.48 | 168 |
| CpG27 | 0.03 | 0.68 | 165 |
| CpG28 | 0.01 | 0.92 | 143 |
| CpG29 | 0.16 | **0.05** | 145 |
| CpG30 | 0.45 | **0.00** | 152 |
| CpG31 | 0.54 | **0.00** | 154 |
| CpG32 | 0.49 | **0.00** | 157 |
| CpG33 | 0.52 | **0.00** | 159 |
| CpG34 | 0.47 | **0.00** | 159 |
| CpG35 | 0.62 | **0.00** | 160 |
| CpG36 | 0.60 | **0.00** | 160 |
| CpG37 | 0.50 | **0.00** | 157 |
| CpG38 | 0.21 | **0.01** | 162 |
| CpG39 | 0.42 | **0.00** | 163 |
| CpG40  Mean | 0.42  0.39 | **0.00**  **0.00** | 163  173 |
|  |  |  |  |

Supplementary table 2. Spearman’s correlations between age and methylation at CpG sites.

|  |  | **Pseudo R squared** | **coefficient** | **SE** | **p-value** | **95% confidence interval** |
| --- | --- | --- | --- | --- | --- | --- |
| **CpG 21** | Dystonia | 0.01 | 0.34 | 0.23 | 0.15 | -0.13 – 0.81 |
|  | Age |  | 0.01 | 0.01 | 0.13 | -0.00 – 0.03 |
| **CpG 22** | Dystonia | 0.01 | 0.45 | 0.48 | 0.36 | -0.51 – 1.40 |
|  | Age |  | 0.00 | 0.01 | 0.70 | -0.01 – 0.01 |
| **CpG 23** | Dystonia | 0.02 | 0.11 | 0.51 | 0.84 | -0.92 – 1.13 |
|  | Age |  | -0.00 | 0.01 | 0.66 | -0.02 – 0.02 |
| **CpG 24** | Dystonia | 0.05 | 1.12 | 0.54 | **0.04** | 0.06 – 2.18 |
|  | Age |  | 0.03 | 0.01 | **0.00** | 0.01 – 0.05 |
| **CpG 25** | Dystonia | 0.00 | -0.08 | 0.79 | 0.92 | -1.63 – 1.48 |
|  | Age |  | -0.01 | 0.02 | 0.65 | -0.04 – 0.02 |
| **CpG 26** | Dystonia | 0.01 | 0.55 | 0.44 | 0.22 | -0.32 – 1.43 |
|  | Age |  | 0.00 | 0.01 | 0.78 | -0.01 – 0.02 |
| **CpG 27** | Dystonia | 0.01 | 0.21 | 0.33 | 0.52 | -0.44 – 0.87 |
|  | Age |  | 0.01 | 0.01 | 0.42 | -0.01 – 0.02 |
| **CpG 28** | Dystonia | 0.01 | 0.21 | 0.41 | 0.61 | -0.59 – 1.01 |
|  | Age |  | 0.01 | 0.01 | 0.30 | -0.01 – 0.03 |
| **CpG 29** | Dystonia | 0.02 | -0.01 | 0.90 | 0.99 | -1.79 – 1.77 |
|  | Age |  | 0.03 | 0.02 | 0.13 | -0.01 – 0.07 |
| **CpG 30** | Dystonia | 0.12 | 0.10 | 0.13 | 0.44 | -0.16 – 0.37 |
|  | Age |  | 0.02 | 0.00 | **0.00** | 0.01 – 0.03 |
| **CpG 31** | Dystonia | 0.21 | 0.04 | 0.11 | 0.70 | -0.17 – 0.26 |
|  | Age |  | 0.02 | 0.00 | **0.00** | 0.02 – 0.03 |
| **CpG 32** | Dystonia | 0.14 | 0.37 | 0.16 | **0.03** | 0.05 – 0.70 |
|  | Age |  | 0.03 | 0.01 | **0.00** | 0.02 – 0.04 |
| **CpG 33** | Dystonia | 0.19 | 0.06 | 0.09 | 0.56 | -0.13 – 0.24 |
|  | Age |  | 0.02 | 0.00 | **0.00** | 0.02 – 0.02 |
| **CpG 34** | Dystonia | 0.16 | 0.15 | 0.12 | 0.19 | -0.07 – 0.37 |
|  | Age |  | 0.02 | 0.00 | **0.00** | 0.01 – 0.03 |
| **CpG 35** | Dystonia | 0.25 | 0.20 | 0.11 | 0.08 | -0.03 – 0.42 |
|  | Age |  | 0.03 | 0.00 | **0.00** | 0.03 – 0.04 |
| **CpG 36** | Dystonia | 0.20 | -0.03 | 0.26 | 0.92 | -0.54 – 0.49 |
|  | Age |  | 0.04 | 0.01 | **0.00** | 0.03 – 0.05 |
| **CpG 37** | Dystonia | 0.11 | 0.03 | 0.14 | 0.82 | -0.25 – 0.31 |
|  | Age |  | 0.03 | 0.00 | **0.00** | 0.02 – 0.04 |
| **CpG 38** | Dystonia | 0.01 | 0.00 | 0.17 | 1.00 | -0.33 – 0.33 |
|  | Age |  | 0.00 | 0.00 | 0.24 | - 0.00 – 0.01 |
| **CpG 39** | Dystonia | 0.10 | 0.09 | 0.13 | 0.52 | -0.18 – 0.35 |
|  | Age |  | 0.02 | 0.00 | **0.00** | 0.01 – 0.03 |
| **CpG 40** | Dystonia | 0.09 | 0.06 | 0.19 | 0.75 | -0.32 – 0.44 |
|  | Age |  | 0.02 | 0.01 | **0.00** | 0.01 – 0.03 |
| **Mean** | Dystonia | 0.06 | 0.12 | 0.17 | 0.48 | -0.21 – 0.45 |
|  | Age |  | 0.02 | 0.00 | **0.00** | 0.01 – 0.03 |

Supplementary table 3. Results of Bootstrapped Quantile regression analysis in all participants with methylation rate per CpG site (including mean) as dependent variables and dystonia and age as independent variables.

|  |  | **Pseudo R squared** | **Coefficient** | **SE** | **p-value** | **95% confidence interval** |
| --- | --- | --- | --- | --- | --- | --- |
| **CpG 21** | Group | 0.03 |  |  |  |  |
|  | CD |  | 0.39 | 0.27 | 0.15 | -0.14 – 0.92 |
|  | DRD |  | 1.00 | 0.47 | 0.04 | 0.07 – 1.93 |
|  | M-D |  | 0.15 | 0.27 | 0.59 | -0.39 – 0.69 |
|  | Age |  | 0.01 | 0.01 | 0.18 | -0.00 – 0.02 |
| **CpG 22** | Group | 0.01 |  |  |  |  |
|  | CD |  | 0.23 | 0.55 | 0.68 | -0.85 – 1.31 |
|  | DRD |  | 0.81 | 0.67 | 0.23 | -0.52 – 2.14 |
|  | M-D |  | 0.32 | 0.52 | 0.54 | -0.71 – 1.36 |
|  | Age |  | 0.01 | 0.01 | 0.49 | -0.01 – 0.02 |
| **CpG 23** | Group | 0.01 |  |  |  |  |
|  | CD |  | 0.03 | 0.53 | 0.96 | -1.02 – 1.08 |
|  | DRD |  | 0.63 | 0.68 | 0.36 | -0.72 – 1.98 |
|  | M-D |  | 0.04 | 0.56 | 0.95 | -1.06 – 1.14 |
|  | Age |  | -0.01 | 0.01 | 0.61 | -0.03 – 0.02 |
| **CpG 24** | Group | 0.06 |  |  |  |  |
|  | CD |  | 0.95 | 0.62 | 0.13 | -0.27 – 2.17 |
|  | DRD |  | 1.73 | 0.66 | 0.01 | 0.43 – 3.04 |
|  | M-D |  | 1.12 | 0.60 | 0.06 | -0.06 – 2.30 |
|  | Age |  | 0.03 | 0.01 | 0.00 | 0.01 – 0.05 |
| **CpG 25** | Group | 0.01 |  |  |  |  |
|  | CD |  | 0.28 | 0.87 | 0.75 | -1.44 – 2.01 |
|  | DRD |  | -0.13 | 0.92 | 0.89 | -1.94 – 1.68 |
|  | M-D |  | -0.74 | 0.82 | 0.37 | -2.35 – 0.87 |
|  | Age |  | -0.00 | 0.02 | 0.96 | -0.03 – 0.03 |
| **CpG 26** | Group | 0.01 |  |  |  |  |
|  | CD |  | 0.21 | 0.55 | 0.70 | -0.87 – 1.29 |
|  | DRD |  | 1.12 | 0.65 | 0.09 | -0.17 – 2.40 |
|  | M-D |  | 0.55 | 0.49 | 0.26 | -0.42 – 1.52 |
|  | Age |  | 0.00 | 0.01 | 0.79 | -0.02 – 0.02 |
| **CpG 27** | Group | 0.02 |  |  |  |  |
|  | CD |  | 0.26 | 0.36 | 0.47 | -0.45 – 0.96 |
|  | DRD |  | 0.74 | 0.51 | 0.15 | -0.26 – 1.74 |
|  | M-D |  | -0.09 | 0.43 | 0.83 | -0.93 – 0.75 |
|  | Age |  | 0.01 | 0.01 | 0.20 | -0.01 – 0.02 |
| **CpG 28** | Group | 0.02 |  |  |  |  |
|  | CD |  | 0.48 | 0.50 | 0.34 | -0.51 – 1.46 |
|  | DRD |  | 0.35 | 0.51 | 0.49 | -0.66 – 1.37 |
|  | M-D |  | -0.19 | 0.52 | 0.71 | -1.21 – 0.83 |
|  | Age |  | 0.01 | 0.01 | 0.37 | -0.01 – 0.03 |
| **CpG 29** | Group | 0.03 |  |  |  |  |
|  | CD |  | -0.47 | 1.05 | 0.66 | -2.54 – 1.60 |
|  | DRD |  | 0.88 | 1.42 | 0.54 | -1.93 – 3.70 |
|  | M-D |  | -0.26 | 1.16 | 0.83 | -2.56 – 2.05 |
|  | Age |  | 0.04 | 0.02 | **0.04** | 0.00 – 0.09 |
| **CpG 30** | Group | 0.13 |  |  |  |  |
|  | CD |  | 0.10 | 0.16 | 0.53 | -0.22 – 0.43 |
|  | DRD |  | 0.10 | 0.22 | 0.64 | -0.33 – 0.53 |
|  | M-D |  | 0.25 | 0.20 | 0.22 | -0.15 – 0.65 |
|  | Age |  | 0.02 | 0.00 | **0.00** | 0.01 – 0.03 |
| **CpG 31** | Group | 0.23 |  |  |  |  |
|  | CD |  | -0.05 | 0.10 | 0.62 | -0.26 – 0.15 |
|  | DRD |  | 0.22 | 0.15 | 0.15 | -0.08 – 0.52 |
|  | M-D |  | 0.15 | 0.14 | 0.26 | -0.11 – 0.42 |
|  | Age |  | 0.02 | 0.00 | **0.00** | 0.02 – 0.03 |
| **CpG 32** | Group | 0.15 |  |  |  |  |
|  | CD |  | 0.32 | 0.18 | 0.07 | -0.03 – 0.67 |
|  | DRD |  | 0.44 | 0.30 | 0.15 | -0.16 – 1.03 |
|  | M-D |  | 0.36 | 0.26 | 0.17 | -0.15 – 0.88 |
|  | Age |  | 0.03 | 0.01 | **0.00** | 0.02 – 0.04 |
| **CpG 33** | Group | 0.19 |  |  |  |  |
|  | CD |  | 0.04 | 0.14 | 0.80 | -0.24 – 0.32 |
|  | DRD |  | 0.02 | 0.15 | 0.91 | -0.27 – 0.31 |
|  | M-D |  | 0.09 | 0.11 | 0.40 | -0.12 – 0.31 |
|  | Age |  | 0.02 | 0.00 | **0.00** | 0.02 – 0.03 |
| **CpG 34** | Group | 0.16 |  |  |  |  |
|  | CD |  | 0.14 | 0.20 | 0.49 | -0.25 – 0.53 |
|  | DRD |  | -0.03 | 0.16 | 0.84 | -0.36 – 0.29 |
|  | M-D |  | 0.20 | 0.12 | 0.10 | -0.04 – 0.43 |
|  | Age |  | 0.02 | 0.00 | **0.00** | 0.01 – 0.03 |
| **CpG 35** | Group | 0.25 |  |  |  |  |
|  | CD |  | 0.18 | 0.13 | 0.16 | -0.07 – 0.43 |
|  | DRD |  | 0.007 | 0.21 | 0.72 | -0.33 – 0.48 |
|  | M-D |  | 0.28 | 0.20 | 0.16 | - 0.11 – 0.67 |
|  | Age |  | 0.03 | 0.00 | **0.00** | 0.02 – 0.04 |
| **CpG 36** | Group | 0.21 |  |  |  |  |
|  | CD |  | -0.02 | 0.31 | 0.94 | -0.63 – 0.58 |
|  | DRD |  | -0.36 | 0.33 | 0.28 | -1.02 – 0.30 |
|  | M-D |  | 0.21 | 0.32 | 0.52 | -0.42 – 0.84 |
|  | Age |  | 0.03 | 0.01 | **0.00** | 0.02 – 0.05 |
| **CpG 37** | Group | 0.11 |  |  |  |  |
|  | CD |  | 0.09 | 0.19 | 0.66 | -0.29 – 0.47 |
|  | DRD |  | -0.07 | 0.23 | 0.76 | -0.52 – 0.38 |
|  | M-D |  | 0.02 | 0.18 | 0.90 | -0.34 – 0.38 |
|  | Age |  | 0.03 | 0.01 | **0.00** | 0.02 – 0.04 |
| **CpG 38** | Group | 0.02 |  |  |  |  |
|  | CD |  | -0.07 | 0.17 | 0.67 | -0.40 – 0.26 |
|  | DRD |  | 0.08 | 0.23 | 0.72 | -0.38 – 0.54 |
|  | M-D |  | 0.08 | 0.26 | 0.77 | -0.44 – 0.59 |
|  | Age |  | 0.00 | 0.00 | 0.37 | - 0.01 – 0.01 |
| **CpG 39** | Group | 0.11 |  |  |  |  |
|  | CD |  | 0.19 | 0.14 | 0.18 | -0.09 – 0.48 |
|  | DRD |  | -0.06 | 0.22 | 0.79 | -0.49 – 0.37 |
|  | M-D |  | 0.06 | 0.24 | 0.81 | -0.42 – 0.53 |
|  | Age |  | 0.02 | 0.00 | **0.00** | 0.01 – 0.03 |
| **CpG 40** | Group | 0.09 |  |  |  |  |
|  | CD |  | 0.08 | 0.23 | 0.71 | -0.36 – 0.53 |
|  | DRD |  | 0.06 | 0.23 | 0.80 | -0.40 – 0.52 |
|  | M-D |  | 0.00 | 0.28 | 0.99 | -0.55 – 0.55 |
|  | Age |  | 0.02 | 0.01 | **0.00** | 0.01 – 0.03 |
| **Mean** | Group | 0.08 |  |  |  |  |
|  | CD |  | -0.01 | 0.18 | 1.00 | -0.35 – 0.35 |
|  | DRD |  | 0.33 | 0.20 | 0.11 | -0.07 – 0.73 |
|  | M-D |  | 0.02 | 0.19 | 0.93 | -0.36 – 0.39 |
|  | Age |  | 0.02 | 0.00 | **0.00** | 0.01 – 0.03 |

Supplementary table 4. Results of Bootstrapped Quantile regression analysis in all participants with methylation rate per CpG site (including mean) as dependent variables and group and age as independent variables. The variable ‘Group’ was entered as dummy variables, with the healthy controls as a reference. Abbreviations used: CD: cervical dystonia, DRD: dopa-responsive dystonia, M-D: myoclonus-dystonia.

|  |  | **Pseudo R squared** | **coefficient** | **SE** | **p-value** | **95% confidence interval** |
| --- | --- | --- | --- | --- | --- | --- |
| **CpG 21** | Group | 0.07 |  |  |  |  |
|  | CD |  | 0.55 | 0.27 | **0.05** | 0.01 – 1.08 |
|  | DRD |  | 1.30 | 0.52 | **0.01** | 0.28 – 2.32 |
|  | M-D |  | 0.19 | 0.22 | 0.39 | -0.25 – 0.63 |
|  | Age |  | 0.01 | 0.01 | 0.22 | -0.01 – 0.02 |
| **CpG 22** | Group | 0.02 |  |  |  |  |
|  | CD |  | 0.30 | 0.60 | 0.62 | -0.90 – 1.50 |
|  | DRD |  | 1.23 | 0.85 | 0.15 | -0.46 – 2.92 |
|  | M-D |  | 0.03 | 0.52 | 0.96 | -1.00 – 1.05 |
|  | Age |  | 0.00 | 0.01 | 0.69 | 0.03 – 0.03 |
| **CpG 23** | Group | 0.04 |  |  |  |  |
|  | CD |  | -0.04 | 0.65 | 0.95 | -1.33 – 1.24 |
|  | DRD |  | 1.44 | 0.71 | **0.05** | 0.04 – 2.85 |
|  | M-D |  | -0.37 | 0.54 | 0.50 | -1.45 – 0.71 |
|  | Age |  | 0.02 | 0.01 | 0.25 | -0.01 – 0.05 |
| **CpG 24** | Group | 0.08 |  |  |  |  |
|  | CD |  | 1.25 | 0.61 | **0.04** | 0.05 – 2.46 |
|  | DRD |  | 2.46 | 0.82 | **0.00** | 0.84 – 4.09 |
|  | M-D |  | 1.03 | 0.60 | 0.09 | -0.16 – 2.21 |
|  | Age |  | 0.02 | 0.01 | **0.05** | 0.00 – 0.05 |
| **CpG 25** | Group | 0.03 |  |  |  |  |
|  | CD |  | 0.70 | 1.12 | 0.53 | -1.52 – 2.93 |
|  | DRD |  | 0.53 | 1.17 | 0.65 | -1.78 – 2.85 |
|  | M-D |  | -0.70 | 1.10 | 0.53 | -2.87 – 1.48 |
|  | Age |  | -0.03 | 0.02 | 0.17 | -0.07 – 0.01 |
| **CpG 26** | Group | 0.02 |  |  |  |  |
|  | CD |  | -0.35 | 0.67 | 0.60 | -1.67 – 0.98 |
|  | DRD |  | 0.67 | 0.75 | 0.37 | -0.82 – 2.17 |
|  | M-D |  | -0.28 | 0.72 | 0.70 | -1.71 – 1.15 |
|  | Age |  | -0.00 | 0.01 | 0.94 | -0.03 – 0.03 |
| **CpG 27** | Group | 0.04 |  |  |  |  |
|  | CD |  | 0.19 | 0.47 | 0.69 | -0.75 – 1.12 |
|  | DRD |  | 0.51 | 0.61 | 0.41 | -0.70 – 1.72 |
|  | M-D |  | -0.59 | 0.49 | 0.23 | -1.57 – 0.39 |
|  | Age |  | 0.00 | 0.01 | 0.57 | -0.01 – 0.02 |
| **CpG 28** | Group | 0.04 |  |  |  |  |
|  | CD |  | 0.60 | 0.50 | 0.23 | -0.39 – 1.60 |
|  | DRD |  | 0.0.7 | 0.66 | 0.92 | -1.24 – 1.38 |
|  | M-D |  | -0.84 | 0.52 | 0.11 | -1.87 – 0.18 |
|  | Age |  | -0.01 | 0.01 | 0.43 | -0.04 – 0.02 |
| **CpG 29** | Group | 0.03 |  |  |  |  |
|  | CD |  | -0.65 | 1.14 | 0.57 | -2.91 – 1.62 |
|  | DRD |  | 0.29 | 1.23 | 0.81 | -2.16 – 2.74 |
|  | M-D |  | -0.30 | 1.33 | 0.82 | -2.94 – 2.33 |
|  | Age |  | 0.05 | 0.03 | 0.11 | -0.01 – 0.11 |
| **CpG 30** | Group | 0.09 |  |  |  |  |
|  | CD |  | 0.24 | 0.20 | 0.25 | -0.17 – 0.64 |
|  | DRD |  | -0.05 | 0.35 | 0.90 | -0.74 – 0.65 |
|  | M-D |  | 0.14 | 0.24 | 0.57 | -0.34 – 0.61 |
|  | Age |  | 0.01 | 0.00 | **0.01** | 0.00 – 0.02 |
| **CpG 31** | Group | 0.18 |  |  |  |  |
|  | CD |  | 0.09 | 0.13 | 0.52 | -0.18 – 0.35 |
|  | DRD |  | 0.15 | 0.24 | 0.53 | -0.32 – 0.63 |
|  | M-D |  | -0.13 | 0.22 | 0.56 | -0.56 – 0.31 |
|  | Age |  | 0.02 | 0.00 | **0.00** | 0.01 – 0.03 |
| **CpG 32** | Group | 0.19 |  |  |  |  |
|  | CD |  | 0.32 | 0.20 | 0.10 | -0.07 – 0.71 |
|  | DRD |  | 0.23 | 0.36 | 0.53 | -0.49 – 0.94 |
|  | M-D |  | 0.32 | 0.43 | 0.46 | -0.53 – 1.17 |
|  | Age |  | 0.03 | 0.01 | **0.00** | 0.02 – 0.05 |
| **CpG 33** | Group | 0.17 |  |  |  |  |
|  | CD |  | 0.03 | 0.15 | 0.84 | -0.27 – 0.33 |
|  | DRD |  | -0.04 | 0.14 | 0.77 | -0.31 – 0.23 |
|  | M-D |  | 0.06 | 0.13 | 0.67 | -0.21 – 0.32 |
|  | Age |  | 0.02 | 0.00 | **0.00** | 0.01 – 0.02 |
| **CpG 34** | Group | 0.13 |  |  |  |  |
|  | CD |  | 0.16 | 0.22 | 0.48 | -0.28 – 0.60 |
|  | DRD |  | 0.19 | 0.21 | 0.39 | -0.24 – 0.61 |
|  | M-D |  | 0.15 | 0.16 | 0.36 | -0.17 – 0.47 |
|  | Age |  | 0.02 | 0.00 | **0.00** | 0.01 – 0.03 |
| **CpG 35** | Group | 0.24 |  |  |  |  |
|  | CD |  | 0.23 | 0.18 | 0.22 | -0.13 – 0.59 |
|  | DRD |  | -0.14 | 0.24 | 0.56 | -0.62 – 0.34 |
|  | M-D |  | 0.02 | 0.27 | 0.95 | - 0.52 – 0.55 |
|  | Age |  | 0.03 | 0.01 | **0.00** | 0.02 – 0.04 |
| **CpG 36** | Group | 0.18 |  |  |  |  |
|  | CD |  | 0.17 | 0.31 | 0.60 | -0.46 – 0.79 |
|  | DRD |  | -0.10 | 0.38 | 0.79 | -0.84 – 0.65 |
|  | M-D |  | 0.03 | 0.42 | 0.95 | -0.80 – 0.85 |
|  | Age |  | 0.04 | 0.01 | **0.00** | 0.02 – 0.05 |
| **CpG 37** | Group | 0.09 |  |  |  |  |
|  | CD |  | 0.12 | 0.22 | 0.59 | -0.32 – 0.56 |
|  | DRD |  | 0.14 | 0.29 | 0.63 | -0.44 – 0.73 |
|  | M-D |  | 0.05 | 0.30 | 0.86 | -0.53 – 0.64 |
|  | Age |  | 0.02 | 0.01 | **0.00** | 0.01 – 0.04 |
| **CpG 38** | Group | 0.01 |  |  |  |  |
|  | CD |  | -0.02 | 0.20 | 0.93 | -0.42 – 0.38 |
|  | DRD |  | 0.17 | 0.27 | 0.54 | -0.37 – 0.71 |
|  | M-D |  | 0.08 | 0.28 | 0.79 | -0.49 – 0.64 |
|  | Age |  | 0.01 | 0.01 | 0.43 | - 0.01 – 0.02 |
| **CpG 39** | Group | 0.12 |  |  |  |  |
|  | CD |  | 0.18 | 0.17 | 0.29 | -0.16 – 0.52 |
|  | DRD |  | -0.03 | 0.29 | 0.93 | -0.61 – 0.56 |
|  | M-D |  | 0.02 | 0.30 | 0.94 | -0.57 – 0.62 |
|  | Age |  | 0.02 | 0.01 | **0.00** | 0.01 – 0.03 |
| **CpG 40** | Group | 0.11 |  |  |  |  |
|  | CD |  | 0.24 | 0.22 | 0.30 | -0.21 – 0.68 |
|  | DRD |  | 0.27 | 0.27 | 0.32 | -0.27 – 0.80 |
|  | M-D |  | -0.31 | 036 | 0.39 | -1.01 – 0.40 |
|  | Age |  | 0.02 | 0.01 | **0.00** | 0.01 – 0.04 |
| **Mean** | Group | 0.10 |  |  |  |  |
|  | CD |  | 0.27 | 0.25 | 0.28 | -0.22 – 0.76 |
|  | DRD |  | 0.44 | 0.29 | 0.12 | -0.12 – 1.01 |
|  | M-D |  | -0.16 | 0.27 | 0.54 | -0.69 – 0.37 |
|  | Age |  | 0.02 | 0.01 | **0.00** | 0.01 – 0.03 |

Supplementary table 5. Results of Bootstrapped Quantile regression analysis in female participants with methylation rate per CpG site as dependent variable and group and age as independent variables. The variable ‘Group’ was entered as dummy variables, with the healthy controls as a reference. Abbreviations used: CD: cervical dystonia, DRD: dopa-responsive dystonia, M-D: myoclonus-dystonia.

|  |  | **Pseudo R squared** | **coefficient** | **SE** | **p-value** | **95% confidence interval** |
| --- | --- | --- | --- | --- | --- | --- |
| **CpG 21** | Dystonia | 0.02 | 0.35 | 0.23 | 0.13 | -0.10 – 0.79 |
|  | Age |  | 0.01 | 0.01 | 0.27 | -0.01 – 0.03 |
| **CpG 22** | Dystonia | 0.01 | 0.27 | 0.55 | 0.63 | -0.83 – 1.36 |
|  | Age |  | 0.01 | 0.01 | 0.43 | -0.01 – 0.02 |
| **CpG 23** | Dystonia | 0.00 | 0.03 | 0.51 | 0.95 | -0.99 – 1.05 |
|  | Age |  | 0.01 | 0.02 | 0.56 | -0.02 – 0.04 |
| **CpG 24** | Dystonia | 0.06 | 1.21 | 0.49 | **0.01** | 0.25 – 2.19 |
|  | Age |  | 0.02 | 0.01 | **0.02** | 0.00 – 0.05 |
| **CpG 25** | Dystonia | 0.00 | -0.24 | 1.06 | 0.82 | -2.33 – 1.85 |
|  | Age |  | -0.01 | 0.02 | 0.48 | -0.05 – 0.02 |
| **CpG 26** | Dystonia | 0.00 | -0.06 | 0.59 | 0.92 | -1.22 – 1.10 |
|  | Age |  | -0.01 | 0.01 | 0.55 | -0.03 – 0.02 |
| **CpG 27** | Dystonia | 0.00 | 0.08 | 0.47 | 0.86 | -0.85 – 1.02 |
|  | Age |  | 0.01 | 0.01 | 0.33 | -0.01 – 0.03 |
| **CpG 28** | Dystonia | 0.00 | 0.11 | 0.44 | 0.80 | -0.76 – 0.98 |
|  | Age |  | -0.00 | 0.01 | 0.77 | -0.02 – 0.02 |
| **CpG 29** | Dystonia | 0.02 | -0.37 | 0.93 | 0.70 | -2.21 – 1.48 |
|  | Age |  | 0.04 | 0.03 | 0.13 | -0.01 – 0.09 |
| **CpG 30** | Dystonia | 0.09 | 0.14 | 0.16 | 0.38 | -0.18 – 0.47 |
|  | Age |  | 0.02 | 0.00 | **0.00** | 0.01 – 0.03 |
| **CpG 31** | Dystonia | 0.18 | 0.09 | 0.14 | 0.54 | -0.19 – 0.37 |
|  | Age |  | 0.02 | 0.00 | **0.00** | 0.01 – 0.03 |
| **CpG 32** | Dystonia | 0.18 | 0.34 | 0.17 | **0.05** | 0.00 – 0.68 |
|  | Age |  | 0.03 | 0.01 | **0.00** | 0.02 – 0.04 |
| **CpG 33** | Dystonia | 0.17 | 0.03 | 0.11 | 0.78 | -0.18 – 0.24 |
|  | Age |  | 0.02 | 0.00 | **0.00** | 0.01 – 0.02 |
| **CpG 34** | Dystonia | 0.13 | 0.16 | 0.13 | 0.24 | -0.11 – 0.42 |
|  | Age |  | 0.02 | 0.00 | **0.00** | 0.01 – 0.03 |
| **CpG 35** | Dystonia | 0.22 | 0.06 | 0.15 | 0.70 | -0.24 – 0.35 |
|  | Age |  | 0.03 | 0.00 | **0.00** | 0.02 – 0.04 |
| **CpG 36** | Dystonia | 0.17 | 0.07 | 0.30 | 0.81 | -0.52 – 0.66 |
|  | Age |  | 0.04 | 0.01 | **0.00** | 0.03 – 0.05 |
| **CpG 37** | Dystonia | 0.09 | 0.10 | 0.17 | 0.56 | -0.24 – 0.43 |
|  | Age |  | 0.02 | 0.01 | **0.00** | 0.01 – 0.03 |
| **CpG 38** | Dystonia | 0.00 | 0.01 | 0.19 | 0.97 | -0.38 – 0.39 |
|  | Age |  | 0.01 | 0.01 | 0.37 | -0.01 – 0.02 |
| **CpG 39** | Dystonia | 0.11 | 0.02 | 0.17 | 0.92 | -0.33 – 0.36 |
|  | Age |  | 0.02 | 0.01 | **0.00** | 0.01 – 0.03 |
| **CpG 40** | Dystonia | 0.10 | 0.17 | 0.20 | 0.40 | -0.23 – 0.57 |
|  | Age |  | 0.02 | 0.01 | **0.01** | 0.01 – 0.03 |
| **Mean** | Dystonia | 0.07 | 0.19 | 0.20 | 0.33 | -0.20 – 0.58 |
|  | Age |  | 0.02 | 0.01 | **0.00** | 0.01 – 0.03 |

Supplementary table 6. Results of Bootstrapped Quantile regression analysis in the female participants with methylation rate per CpG site as dependent variable and dystonia and age as independent variables.

|  |  | **Pseudo R squared** | **coefficient** | **SE** | **p-value** | **95% confidence interval** |
| --- | --- | --- | --- | --- | --- | --- |
| **CpG 21** | Group | 0.02 |  |  |  |  |
|  | CD |  | -0.51 | 0.73 | 0.49 | -1.97 – 0.95 |
|  | DRD |  | -0.67 | 0.91 | 0.46 | -2.50 – 1.15 |
|  | M-D |  | -0.11 | 0.75 | 0.89 | -1.60 – 1.39 |
|  | Age |  | -0.00 | 0.01 | 0.69 | -0.03 – 0.02 |
| **CpG 22** | Group | 0.02 |  |  |  |  |
|  | CD |  | -0.20 | 1.40 | 0.89 | -3.00 – 2.61 |
|  | DRD |  | 0.44 | 1.72 | 0.80 | -3.01 – 3.89 |
|  | M-D |  | 0.82 | 1.53 | 0.60 | -2.27 – 3.89 |
|  | Age |  | -0.00 | 0.01 | 0.94 | -0.03 – 0.03 |
| **CpG 23** | Group | 0.04 |  |  |  |  |
|  | CD |  | 0.04 | 1.90 | 0.99 | -3.78 – 3.85 |
|  | DRD |  | 0.87 | 1.97 | 0.66 | -3.09 – 4.83 |
|  | M-D |  | 1.57 | 1.95 | 0.43 | -2.36 – 5.50 |
|  | Age |  | -0.02 | 0.01 | 0.18 | -0.05 – 0.01 |
| **CpG 24** | Group | 0.04 |  |  |  |  |
|  | CD |  | -0.48 | 2.48 | 0.85 | -5.46 – 4.51 |
|  | DRD |  | 0.68 | 2.48 | 0.78 | -4.30 – 5.66 |
|  | M-D |  | 0.60 | 2.69 | 0.83 | -4.81 – 6.00 |
|  | Age |  | 0.03 | 0.03 | 0.34 | -0.03 – 0.08 |
| **CpG 25** | Group | 0.06 |  |  |  |  |
|  | CD |  | -0.62 | 1.09 | 0.57 | -2.82 – 1.58 |
|  | DRD |  | 0.10 | 1.04 | 0.92 | -2.01 – 2.21 |
|  | M-D |  | 0.28 | 1.09 | 0.80 | -1.91 – 2.47 |
|  | Age |  | 0.03 | 0.02 | 0.11 | -0.01 – 0.07 |
| **CpG 26** | Group | 0.05 |  |  |  |  |
|  | CD |  | 1.42 | 1.01 | 0.16 | -0.60 – 3.44 |
|  | DRD |  | 0.31 | 1.15 | 0.79 | -1.99 – 2.61 |
|  | M-D |  | 1.21 | 0.74 | 0.11 | -0.27 – 2.68 |
|  | Age |  | 0.00 | 0.01 | 0.73 | -0.02 – 0.03 |
| **CpG 27** | Group | 0.04 |  |  |  |  |
|  | CD |  | -0.21 | 0.87 | 0.81 | -1.95 – 1.53 |
|  | DRD |  | -0.16 | 1.05 | 0.88 | -2.26 – 1.94 |
|  | M-D |  | 0.56 | 0.63 | 0.38 | -0.71 – 1.84 |
|  | Age |  | 0.00 | 0.01 | 0.92 | -0.02 – 0.03 |
| **CpG 28** | Group | 0.10 |  |  |  |  |
|  | CD |  | 1.30 | 1.29 | 0.32 | -1.30 – 3.90 |
|  | DRD |  | 0.17 | 1.11 | 0.88 | -2.08 – 2.41 |
|  | M-D |  | 0.19 | 0.84 | 0.83 | -1.51 – 1.88 |
|  | Age |  | 0.03 | 0.01 | 0.05 | -0.00 – 0.05 |
| **CpG 29** | Group | 0.08 |  |  |  |  |
|  | CD |  | 0.84 | 2.85 | 0.77 | -4.93 – 6.60 |
|  | DRD |  | 5.94 | 3.83 | 0.13 | -1.81 – 13.68 |
|  | M-D |  | 0.98 | 2.91 | 0.74 | -4.91 – 6.87 |
|  | Age |  | 0.06 | 0.04 | 0.14 | -0.02 – 0.13 |
| **CpG 30** | Group | 0.32 |  |  |  |  |
|  | CD |  | -0.46 | 0.27 | 0.10 | -1.00 – 0.09 |
|  | DRD |  | -0.26 | 0.33 | 0.44 | -9.28 – 0.42 |
|  | M-D |  | -0.07 | 0.28 | 0.81 | -0.62 – 0.49 |
|  | Age |  | 0.03 | 0.00 | **0.00** | 0.02 – 0.03 |
| **CpG 31** | Group | 0.41 |  |  |  |  |
|  | CD |  | -0.46 | 0.29 | 0.12 | -1.04 – 0.12 |
|  | DRD |  | 0.14 | 0.29 | 0.64 | -0.45 – 0.73 |
|  | M-D |  | 0.05 | 0.31 | 0.88 | -0.59 – 0.68 |
|  | Age |  | 0.03 | 0.00 | **0.00** | 0.02 – 0.04 |
| **CpG 32** | Group | 0.12 |  |  |  |  |
|  | CD |  | -0.15 | 0.45 | 0.73 | -1.06 – 0.75 |
|  | DRD |  | 0.38 | 0.57 | 0.51 | -0.77 – 1.53 |
|  | M-D |  | 0.13 | 0.44 | 0.77 | -0.76 – 1.02 |
|  | Age |  | 0.03 | 0.01 | **0.02** | 0.01 – 0.05 |
| **CpG 33** | Group | 0.25 |  |  |  |  |
|  | CD |  | 0.06 | 0.38 | 0.88 | -0.71 – 0.83 |
|  | DRD |  | 0.07 | 0.40 | 0.86 | -0.73 – 0.87 |
|  | M-D |  | 0.23 | 0.31 | 0.46 | -0.39 – 0.86 |
|  | Age |  | 0.03 | 0.01 | **0.00** | 0.02 – 0.04 |
| **CpG 34** | Group | 0.26 |  |  |  |  |
|  | CD |  | 0.19 | 0.36 | 0.59 | -0.52 – 0.91 |
|  | DRD |  | -0.42 | 0.35 | 0.23 | -1.12 – 0.28 |
|  | M-D |  | 0.10 | 0.30 | 0.74 | -0.51 – 0.71 |
|  | Age |  | 0.01 | 0.01 | **0.02** | 0.00 – 0.03 |
| **CpG 35** | Group | 0.36 |  |  |  |  |
|  | CD |  | 0.10 | 0.27 | 0.72 | -0.44 – 0.64 |
|  | DRD |  | 0.12 | 0.30 | 0.69 | -0.49 – 0.73 |
|  | M-D |  | 0.55 | 0.33 | 0.11 | -0.13 – 1.23 |
|  | Age |  | 0.03 | 0.01 | **0.00** | 0.01 – 0.04 |
| **CpG 36** | Group | 0.35 |  |  |  |  |
|  | CD |  | -0,41 | 0.53 | 0.44 | -1.47 – 0.65 |
|  | DRD |  | -0.73 | 0.57 | 0.20 | -1.87 – 0.41 |
|  | M-D |  | -0.28 | 0.49 | 0.57 | -1.26 – 0.70 |
|  | Age |  | 0.04 | 0.01 | **0.00** | 0.02 – 0.06 |
| **CpG 37** | Group | 0.23 |  |  |  |  |
|  | CD |  | -0.26 | 0.47 | 0.57 | -1.20 – 0.67 |
|  | DRD |  | -0.82 | 0.61 | 0.19 | -2.05 – 0.41 |
|  | M-D |  | -0.52 | 0.47 | 0.27 | -1.47 – 0.42 |
|  | Age |  | 0.03 | 0.01 | **0.02** | 0.00 – 0.05 |
| **CpG 38** | Group | 0.06 |  |  |  |  |
|  | CD |  | -0.37 | 0.31 | 0.24 | -0.99 – 0.26 |
|  | DRD |  | 0.24 | 0.45 | 0.60 | -0.67 – 1.15 |
|  | M-D |  | 0.31 | 0.42 | 0.46 | -0.53 – 1.16 |
|  | Age |  | 0.02 | 0.01 | **0.04** | 0.00 – 0.03 |
| **CpG 39** | Group | 0.11 |  |  |  |  |
|  | CD |  | -0.07 | 0.28 | 0.80 | -0.64 – 0.49 |
|  | DRD |  | -0.05 | 0.41 | 0.91 | -0.87 – 0.78 |
|  | M-D |  | 0.07 | 0.33 | 0.82 | -0.59 – 0.74 |
|  | Age |  | 0.01 | 0.01 | 0.08 | -0.00 – 0.03 |
| **CpG 40** | Group | 0.14 |  |  |  |  |
|  | CD |  | 0.20 | 0.40 | 0.62 | -0.60 – 1.00 |
|  | DRD |  | -0.02 | 0.46 | 0.97 | -0.94 – 0.90 |
|  | M-D |  | 0.50 | 0.44 | 0.27 | -0.40 – 1.39 |
|  | Age |  | 0.01 | 0.01 | 0.07 | -0.00 – 0.03 |
| **Mean** | Group | 0.11 |  |  |  |  |
|  | CD |  | -0.18 | 0.41 | 0.67 | -1.01 – 0.65 |
|  | DRD |  | 0.18 | 0.53 | 0.74 | -0.88 – 1.23 |
|  | MD |  | 0.18 | 0.47 | 0.70 | -0.76 – 1.11 |
|  | Age |  | 0.02 | 0.01 | **0.02** | 0.00 – 0.03 |

Supplementary table 7. Results of Bootstrapped Quantile regression analysis in male participants with methylation rate per CpG site as dependent variable and group and age as independent variables. The variable ‘Group’ was entered as dummy variables, with the healthy controls as a reference. Abbreviations used: CD: cervical dystonia, DRD: dopa-responsive dystonia, M-D: myoclonus-dystonia.

|  |  | **Pseudo R squared** | **coefficient** | **SE** | **p-value** | **95% confidence interval** |
| --- | --- | --- | --- | --- | --- | --- |
| **CpG 21** | Dystonia | 0.01 | -0.48 | 0.79 | 0.55 | -2.07 – 1.11 |
|  | Age |  | 0.00 | 0.01 | 0.96 | -0.02 – 0.02 |
| **CpG 22** | Dystonia | 0.01 | 0.44 | 1.50 | 0.77 | -2.57 – 3.45 |
|  | Age |  | -0.00 | 0.01 | 0.68 | -0.03 – 0.02 |
| **CpG 23** | Dystonia | 0.01 | 0.75 | 1.96 | 0.70 | -3.18 – 4.69 |
|  | Age |  | -0.02 | 0.01 | 0.14 | -0.05 – 0.01 |
| **CpG 24** | Dystonia | 0.04 | 0.68 | 2.32 | 0.77 | -3.98 – 5.34 |
|  | Age |  | 0.02 | 0.02 | 0.18 | -0.01 – 0.05 |
| **CpG 25** | Dystonia | 0.05 | 0.22 | 0.93 | 0.81 | -1.64 – 2.08 |
|  | Age |  | 0.03 | 0.02 | 0.07 | -0.00 – 0.07 |
| **CpG 26** | Dystonia | 0.04 | 1.01 | 0.64 | 0.12 | -0.27 – 2.30 |
|  | Age |  | 0.00 | 0.01 | 0.98 | -0.03 – 0.03 |
| **CpG 27** | Dystonia | 0.02 | 0.33 | 0.66 | 0.62 | -0.99 – 1.65 |
|  | Age |  | 0.01 | 0.01 | 0.44 | -0.02 – 0.04 |
| **CpG 28** | Dystonia | 0.05 | 0.19 | 0.89 | 0.83 | -1.61 – 1.99 |
|  | Age |  | 0.03 | 0.01 | 0.08 | -0.00 – 0.06 |
| **CpG 29** | Dystonia | 0.03 | 2.30 | 2.72 | 0.40 | -3.18 – 7.78 |
|  | Age |  | 0.02 | 0.03 | 0.50 | -0.04 – 0.09 |
| **CpG 30** | Dystonia | 0.27 | -0.25 | 0.26 | 0.35 | -0.77 – 0.28 |
|  | Age |  | 0.02 | 0.00 | **0.00** | 0.01 – 0.03 |
| **CpG 31** | Dystonia | 0.32 | -0.22 | 0.29 | 0.45 | -0.80 – 0.36 |
|  | Age |  | 0.02 | 0.00 | **0.00** | 0.02 – 0.03 |
| **CpG 32** | Dystonia | 0.11 | -0.04 | 0.37 | 0.92 | -0.79 – 0.72 |
|  | Age |  | 0.02 | 0.01 | **0.00** | 0.01 – 0.04 |
| **CpG 33** | Dystonia | 0.25 | 0.23 | 0.31 | 0.45 | -0.38 – 0.85 |
|  | Age |  | 0.03 | 0.00 | **0.00** | 0.02 – 0.04 |
| **CpG 34** | Dystonia | 0.21 | 0.15 | 0.29 | 0.60 | -0.43 – 0.73 |
|  | Age |  | 0.03 | 0.00 | **0.00** | 0.02 – 0.04 |
| **CpG 35** | Dystonia | 0.34 | 0.16 | 0.24 | 0.51 | -0.32 – 0.64 |
|  | Age |  | 0.03 | 0.00 | **0.00** | 0.02 – 0.04 |
| **CpG 36** | Dystonia | 0.33 | -0.42 | 0.44 | 0.34 | -1.30 – 0.45 |
|  | Age |  | 0.05 | 0.01 | **0.00** | 0.03 – 0.06 |
| **CpG 37** | Dystonia | 0.22 | -0.45 | 0.41 | 0.29 | -1.28 – 0.39 |
|  | Age |  | 0.03 | 0.01 | **0.00** | 0.02 – 0.05 |
| **CpG 38** | Dystonia | 0.04 | -0.07 | 0.30 | 0.82 | -0.67 – 0.54 |
|  | Age |  | 0.01 | 0.01 | 0.33 | -0.01 – 0.02 |
| **CpG 39** | Dystonia | 0.10 | 0.02 | 0.23 | 0.95 | -0.44 – 0.47 |
|  | Age |  | 0.02 | 0.01 | **0.02** | 0.00 – 0.03 |
| **CpG 40** | Dystonia | 0.12 | 0.22 | 0.37 | 0.57 | -0.53 – 0.97 |
|  | Age |  | 0.02 | 0.01 | **0.02** | 0.00 – 0.03 |
| **Mean** | Dystonia | 0.07 | -0.06 | 0.41 | 0.88 | -0.88 – 0.75 |
|  | Age |  | 0.02 | 0.01 | **0.02** | 0.00 – 0.03 |

Supplementary table 8. Results of Bootstrapped Quantile regression analysis in the male participants with methylation rate per CpG site as dependent variable and dystonia and age as independent variables.

|  | z-score depression | | z-score anxiety | |
| --- | --- | --- | --- | --- |
|  | *r_s_* | *p-*value | *r_s_* | *p*-  value |
| CpG21 | -0.06 | 0.55 | -0.09 | 0.35 |
| CpG22 | -0.18 | 0.06 | -0.18 | 0.06 |
| CpG23 | -0.08 | 0.40 | -0.10 | 0.31 |
| CpG24 | -0.05 | 0.57 | -0.07 | 0.48 |
| CpG25 | -0.02 | 0.83 | 0.04 | 0.64 |
| CpG26 | 0.00 | 0.99 | 0.10 | 0.33 |
| CpG27 | -0.18 | 0.06 | -0.11 | 0.26 |
| CpG28 | -0.15 | 0.14 | -0.14 | 0.17 |
| CpG29 | -0.08 | 0.41 | -0.08 | 0.42 |
| CpG30 | 0.18 | 0.07 | 0.08 | 0.46 |
| CpG31 | 0.02 | 0.85 | 0.10 | 0.33 |
| CpG32 | 0.10 | 0.32 | 0.07 | 0.48 |
| CpG33 | 0.08 | 0.39 | 0.11 | 0.28 |
| CpG34 | 0.23 | **0.02** | 0.28 | **0.00** |
| CpG35 | 0.00 | 0.99 | 0.19 | 0.29 |
| CpG36 | 0.14 | 0.15 | 0.19 | 0.06 |
| CpG37 | -0.00 | 0.97 | 0.07 | 0.48 |
| CpG38 | -0.15 | 0.13 | -0.20 | **0.04** |
| CpG39 | -0.06 | 0.53 | -0.03 | 0.79 |
| CpG40 | -0.03 | 0.73 | 0.03 | 0.78 |
| Mean | -0.12 | 0.20 | -0.04 | 0.65 |

Supplementary table 9. Spearman’s correlation between methylation at CpG sites (including mean methylation) and severity of psychiatry in whole dystonia group.

Supplementary figure 1. Boxplot of methylation rate (%) at CpG 28 in dystonia patients with and without a psychiatric disorder in their lifetime, according to the MINI-PLUS/KID.

|  | Cervical dystonia | | | | Myoclonus-dystonia | | | | Dopa-responsive dystonia | | | |
| --- | --- | --- | --- | --- | --- | --- | --- | --- | --- | --- | --- | --- |
|  | z-score depression | | z-score anxiety | | z-score depression | | z-score anxiety | | z-score depression | | z-score anxiety | |
|  | *r_s_* | *p-*value | *r_s_* | *p-*value | *r_s_* | *p-*value | *r_s_* | *p-*value | *r_s_* | *p-*value | *r_s_* | *p*-  value |
| CpG21 | -0.11 | 0.47 | -0.17 | 0.25 | -0.03 | 0.88 | -0.08 | 0.61 | -0.05 | 0.81 | 0.10 | 0.64 |
| CpG22 | -0.20 | 0.18 | -0.17 | 0.24 | -0.33 | **0.04** | -0.31 | 0.06 | 0.06 | 0.77 | 0.00 | 0.99 |
| CpG23 | 0.07 | 0.64 | 0.01 | 0.95 | -0.21 | 0.21 | -0.31 | 0.06 | -0.07 | 0.75 | -0.02 | 0.92 |
| CpG24 | -0.15 | 0.30 | -0.09 | 0.56 | -0.06 | 0.73 | -0.09 | 0.57 | 0.20 | 0.34 | 0.11 | 0.59 |
| CpG25 | -0.10 | 0.52 | -0.04 | 0.76 | -0.05 | 0.74 | -0.01 | 0.94 | 0.08 | 0.70 | 0.26 | 0.22 |
| CpG26 | 0.11 | 0.48 | 0.16 | 0.28 | -0.09 | 0.58 | 0.04 | 0.79 | -0.05 | 0.83 | -0.03 | 0.88 |
| CpG27 | -0.12 | 0.43 | -0.05 | 0.75 | -0.31 | 0.06 | -0.20 | 0.24 | -0.32 | 0.13 | -0.25 | 0.25 |
| CpG28 | -0.08 | 0.63 | -0.05 | 0.74 | -0.43 | **0.02** | -0.39 | **0.03** | -0.01 | 0.95 | -0.12 | 0.60 |
| CpG29 | -0.25 | 0.11 | -0.22 | 0.16 | 0.11 | 0.52 | -0.01 | 0.95 | -0.15 | 0.53 | 0.11 | 0.63 |
| CpG30 | 0.13 | 0.38 | -0.06 | 0.70 | -0.01 | 0.97 | 0.19 | 0.31 | 0.50 | **0.02** | -0.11 | 0.62 |
| CpG31 | 0.05 | 0.75 | -0.08 | 0.60 | -0.20 | 0.28 | 0.15 | 0.42 | 0.28 | 0.19 | 0.05 | 0.84 |
| CpG32 | -0.02 | 0.91 | -0.20 | 0.17 | 0.02 | 0.93 | 0.27 | 0.13 | 0.33 | 0.12 | -0.06 | 0.81 |
| CpG33 | -0.01 | 0.96 | -0.04 | 0.81 | -0.26 | 0.15 | 0.16 | 0.37 | 0.55 | **0.01** | 0.01 | 0.97 |
| CpG34 | 0.30 | **0.04** | 0.17 | 0.25 | -0.10 | 0.59 | 0.26 | 0.14 | 0.50 | **0.01** | 0.35 | 0.11 |
| CpG35 | -0.06 | 0.67 | -0.07 | 0.66 | -0.28 | 0.11 | 0.07 | 0.69 | 0.23 | 0.27 | 0.10 | 0.67 |
| CpG36 | 0.08 | 0.61 | 0.07 | 0.65 | -0.06 | 0.72 | 0.21 | 0.23 | 0.35 | 0.10 | 0.23 | 0.30 |
| CpG37 | -0.15 | 0.31 | -0.06 | 0.67 | -0.06 | 0.73 | 0.17 | 0.34 | 0.22 | 0.31 | -0.09 | 0.70 |
| CpG38 | -0.19 | 0.20 | -0.30 | **0.04** | -0.20 | 0.27 | -0.21 | 0.23 | 0.14 | 0.49 | -0.04 | 0.86 |
| CpG39 | 0.04 | 0.76 | -0.14 | 0.33 | -0.26 | 0.14 | 0.02 | 0.91 | 0.07 | 0.72 | -0.06 | 0.78 |
| CpG40 | -0.19 | 0.20 | -0.13 | 0.37 | -0.20 | 0.25 | 0.08 | 0.65 | 0.14 | 0.51 | -0.18 | 0.38 |
| Mean | -0.20 | 0.17 | -0.14 | 0.35 | -0.29 | 0.07 | -0.13 | 0.41 | 0.16 | 0.43 | 0.22 | 0.29 |

Supplementary table 10. Spearman’s correlation between methylation at CpG sites (including mean methylation) and severity of psychiatry per dystonia group.

|  | Cervical dystonia | | | | | | Myoclonus-dystonia | | | | | | Dopa-responsive dystonia | | | | | |
| --- | --- | --- | --- | --- | --- | --- | --- | --- | --- | --- | --- | --- | --- | --- | --- | --- | --- | --- |
|  | Depression | | Anxiety | | Psychiatry | | Depression | | Anxiety | | Psychiatry | | Depression | | Anxiety | | Psychiatry | |
|  | *Yes* | *No* | *Yes* | *No* | *Yes* | *No* | *Yes* | *No* | *Yes* | *No* | *Yes* | *No* | *Yes* | *No* | *Yes* | *No* | *Yes* | *No* |
| CpG21 | 2.5 | 2.9 | 2.6 | 2.7 | 2.5 | 3.0 | 2.3 | 2.4 | 2.3 | 2.3 | 2.3 | 2.9 | 4.2 | 2.9 | 3.8 | 3.2 | 3.4 | 2.7 |
| CpG22 | **2.3** | **3.2** | 3.1 | 2.7 | 2.5 | 3.0 | 2.6 | 2.8 | 2.5 | 3.3 | **2.5** | **3.3** | 3.4 | 3.1 | 5.1 | 2.9 | 5.1 | 2.8 |
| CpG23 | 3.5 | 3.1 | 3.5 | 2.9 | 3.5 | 2.7 | 3.1 | 3.3 | 2.7 | 3.8 | 3.1 | 4.1 | 5.1 | 3.3 | 4.7 | 3.9 | 4.6 | 3.6 |
| CpG24 | 3.7 | 4.6 | 4.4 | 4.1 | 4.1 | 4.7 | 4.3 | 4.3 | 3.5 | 4.6 | 3.5 | 4.9 | 5.5 | 4.6 | 5.2 | 4.9 | 5.4 | 4.5 |
| CpG25 | 4.8 | 5.7 | 5.7 | 4.7 | 5.2 | 4.4 | 4.1 | 4.0 | 3.9 | 4.5 | 4.0 | 4.5 | 6.6 | 4.2 | 5.4 | 4.7 | 5.4 | 4.6 |
| CpG26 | 3.4 | 3.2 | 4.2 | 2.9 | 3.2 | 3.5 | 3.6 | 3.3 | 3.3 | 3.6 | 3.4 | 3.6 | 4.5 | 3.8 | 4.0 | 3.5 | 4.0 | 3.4 |
| CpG27 | 2.5 | 2.5 | 2.7 | 2.5 | 2.5 | 2.6 | 1.6 | 2.5 | **1.6** | **2.8** | **1.6** | **2.9** | 3.7 | 2.3 | 3.2 | 2.6 | 3.2 | 2.1 |
| CpG28 | 4.6 | 4.4 | 4.7 | 4.1 | 4.6 | 4.1 | 3.1 | 4.0 | **3.1** | **4.5** | **3.1** | **4.7** | 4.1 | 4.3 | 4.1 | 4.3 | 4.0 | 4.4 |
| CpG29 | 6.9 | 8.1 | 7.4 | 8.1 | 6.9 | 8.4 | 6.2 | 7.4 | 6.2 | 8.0 | 6.2 | 8.7 | 8.6 | 7.6 | 8.7 | 7.6 | 8.7 | 7.6 |
| CpG30 | 3.1 | 3.3 | 3.3 | 3.2 | 3.2 | 3.5 | 3.3 | 3.1 | 2.9 | 3.4 | 2.9 | 3.4 | 2.7 | 3.1 | 3.1 | 2.7 | 2.9 | 2.8 |
| CpG31 | 2.0 | 2.3 | 2.3 | 2.1 | 2.3 | 2.2 | 2.1 | 2.2 | 2.0 | 2.3 | 2.0 | 2.4 | 1.7 | 1.7 | 2.5 | 1.6 | 1.8 | 1.7 |
| CpG32 | 4.8 | 4.8 | 4.8 | 4.8 | 4.8 | 5.1 | 4.5 | 4.4 | 4.4 | 4.6 | 4.4 | 4.6 | 3.9 | 4.4 | 5.1 | 4.0 | 4.5 | 4.0 |
| CpG33 | 2.0 | 2.2 | 2.0 | 2.2 | 2.0 | 2.3 | 2.1 | 2.1 | 2.1 | 2.2 | 2.1 | 2.3 | 1.7 | 1.7 | 1.7 | 1.5 | 1.8 | 1.5 |
| CpG34 | 3.2 | 2.8 | 3.1 | 2.8 | 3.1 | 2.8 | 3.1 | 2.9 | 3.0 | 2.9 | 2.7 | 3.0 | 2.4 | 2.6 | 2.5 | 2.3 | 2.5 | 2.1 |
| CpG35 | **3.0** | **3.5** | 3.3 | 3.3 | 3.2 | 3.6 | 3.0 | 3.2 | 2.8 | 3.3 | **2.8** | **3.6** | 2.5 | 2.5 | 2.7 | 2.3 | 2.6 | 2.3 |
| CpG36 | 5.1 | 4.9 | 5.2 | 4.9 | 5.0 | 4.9 | 5.2 | 5.0 | 4.9 | 5.2 | 4.9 | 5.3 | 4.1 | 3.9 | 4.3 | 3.9 | 4.2 | 3.9 |
| CpG37 | 4.5 | 4.7 | 4.6 | 4.5 | 4.5 | 4.8 | 4.2 | 4.3 | 4.0 | 4.5 | 4.0 | 4.6 | 3.4 | 4.2 | 4.5 | 3.9 | 4.5 | 3.6 |
| CpG38 | 3.8 | 3.9 | 3.9 | 3.8 | 3.8 | 4.2 | 3.8 | 4.1 | 3.9 | 4.1 | 3.9 | 4.1 | **3.4** | **4.2** | 3.9 | 3.8 | 4.0 | 3.6 |
| CpG39 | 3.3 | 3.3 | 3.3 | 3.3 | 3.3 | 3.5 | 3.0 | 3.3 | 3.1 | 3.4 | 3.0 | 3.5 | 2.7 | 3.0 | 2.8 | 3.1 | 2.8 | 3.2 |
| CpG40 | 3.8 | 3.9 | 3.9 | 3.9 | 3.8 | 4.1 | 3.8 | 3.6 | 3.5 | 3.7 | 3.5 | 3.7 | 3.5 | 3.2 | 3.7 | 3.2 | **3.7** | **2.9** |
| Mean | 3.7 | 3.9 | 4.0 | 3.8 | 3.8 | 4.1 | 3.6 | 4.0 | 3.5 | 4.1 | 3.5 | 4.2 | 4.0 | 3.7 | 4.0 | 3.7 | 4.0 | 3.7 |

Supplementary table 11. Methylation per CpG site in patients with and without a lifetime diagnosis of depression, anxiety or any psychiatric disorder per dystonia subtype. Data is presented as median. Medians depicted in bold are significantly different from each other (*p<*0.05), analysed by Mann Withney U tests.

|  | L_A_/L_A_  n=31 | S/S  n=20 | Other  n=62 | *p-*value |
| --- | --- | --- | --- | --- |
| BDI | 6 (0-20) | 9 (0-22) | 8 (0-30) | 0.39 |
| BAI | 7 (0-42) | 8 (1-31) | 7 (0-32) | 0.43 |
| CDI | 89 (85-107) | 77 (73-117) | 97 (76-126) | 0.67 |
| SCARED | 5 (1-19) | 5 (2-6) | 5 (3-12) | 0.88 |
| Depression | 10 (32%) | 6 (30%) | 21 (50%) | 0.95 |
| Anxiety | 15 (48%) | 7 (35%) | 45 (73%) | 0.50 |
| Any psychiatric disorder | 19 (61%) | 9 (45%) | 45 (73%) | 0.07 |

Supplementary table 12. Psychiatry and genotype of SERT polymorphism. Kruskal Wallis test and χ^2^ tests were performed to compute *p*-values. Abbreviations used: BDI: Beck Depression Inventory (adults only), BAI: Beck Anxiety Inventory (adults only), CDI: Child Depression Inventory (children only), SCARED: Screen for Child Anxiety and Related Emotional Disorders (children only).

| **CpG position** | **Primer sequence** | **Sequence to analyze** |
| --- | --- | --- |
| **CpG 21-27** | Fwd: 5'-GTTTTAGGAAGAAAGAGAGAGTA-3' |  |
|  | Rev: 5'- Biotin-CACTTTAAAACCAATAAACTTAAT-3' |  |
|  | seq: 5'-TTTTTGGTTTTGGGG-3' | TYGGGYGYGTATTTYGTTTYGTAGYGYGGTTTTTTTTTGGYGAGYGTAATTTTATTTAG |
| **CpG 36-28** | Fwd: 5'-GGGGAAGAAGGTTTGGAAAGA-3' |  |
|  | Rev: 5'- Biotin-AAAATCCCTCCCCTCCTA-3' |  |
|  | seq: 5'-TTTGAGGAGAATAAATTTAATGTTT-3' | TTTYGYGGTYGYGGTTTYGYGTTTTYGTTGGATGGGGTTGYGTTYGTTAGGGAGGGGT |
| **CpG 40-37** | Fwd: 5'-GGGGAAGAAGGTTTGGAAAGA-3' |  |
|  | Rev: 5'- Biotin-AAAATCCCTCCCCTCCTA-3' |  |
|  | seq: 5'-TTTTATATGGTTTGATTTTTAGATA-3' | GTYGTYGTTAAAGAGTTTTTGAAGAATTTTTGYGTTATTTTGAGGYGAATAAATTTAATGTTTTTT |

Supplementary table 13. Primer sequences used in quantitative bisulfite pyrosequencing**.**


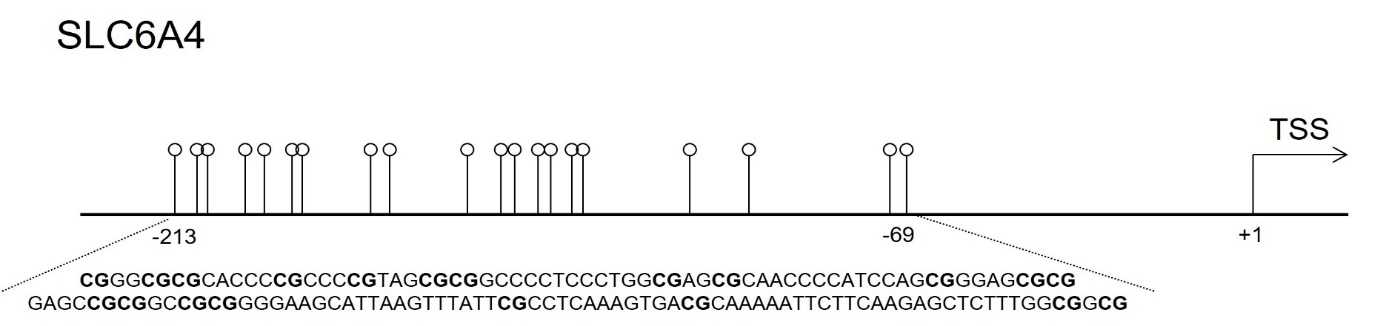


Supplementary figure 2. A schematic diagram for the studied CpG sites in *SLC6A4* promoter region in relation to the transcriptional start site (TSS).

**Supplementary methods 5-HTTLPR genotyping**

Polymerase chain reaction (PCR) with Forward primer FAM-5’-TGAATGCCAGCACCTAACCC-3’ and Reverse primer 5’-TTCTGGTGCCACCTAGACGC-3’ were used to determine L_A_/L_G_/S variants of the promotor region of *SLC6A4.* The PCR product was ingested with Msp-I for at least 3 hours at 37 ºC and the resulting restriction fragments were separated using capillary electrophoresis (ABI 3130 analyze; Applied Biosystems, the Netherlands) and fragments segments were estimated using the ABI Prism ® GeneMapper^TM^ software (Applied Biosystems). The L_A_, L_G_ an S variants were determined by detection of fragments of 325, 284 and 152 base pairs, respectively.
